# A Novel Fault Diagnosis Method Using FCEEMD-Based Multi-Complexity Low-Dimensional Features and Directed Acyclic Graph LSTSVM

**DOI:** 10.3390/e26121031

**Published:** 2024-11-29

**Authors:** Rongrong Lu, Miao Xu, Chengjiang Zhou, Zhaodong Zhang, Kairong Tan, Yuhuan Sun, Yuran Wang, Min Mao

**Affiliations:** 1 School of Information Science and Technology, Yunnan Normal University, Kunming 650500, China; lurongrong_02@163.com (R.L.); miao1xu@163.com (M.X.); zhaodong_zhang618@163.com (Z.Z.); kairongtan@163.com (K.T.); yuhuansun0926@163.com (Y.S.); wyuran_27@163.com (Y.W.); 2 Faculty of Information Engineering, Quzhou College of Technology, Quzhou 324000, China

**Keywords:** fast complementary ensemble empirical mode decomposition, fault feature selection, feature extraction, directed acyclic graph least squares twin support vector machine, fault diagnosis

## Abstract

Rolling bearings, as critical components of rotating machinery, significantly influence equipment reliability and operational efficiency. Accurate fault diagnosis is therefore crucial for maintaining industrial production safety and continuity. This paper presents a new fault diagnosis method based on FCEEMD multi-complexity low-dimensional features and directed acyclic graph LSTSVM. The Fast Complementary Ensemble Empirical Mode Decomposition (FCEEMD) method is applied to decompose vibration signals, effectively reducing background noise. Nonlinear complexity features are then extracted, including sample entropy (SE), permutation entropy (PE), dispersion entropy (DE), Gini coefficient, the square envelope Gini coefficient (SEGI), and the square envelope spectral Gini coefficient (SESGI), enhancing the capture of the signal complexity. In addition, 16 time-domain and 13 frequency-domain features are used to characterize the signal, forming a high-dimensional feature matrix. Robust unsupervised feature selection with local preservation (RULSP) is employed to identify low-dimensional sensitive features. Finally, a multi-classifier based on DAG LSTSVM is constructed using the directed acyclic graph (DAG) strategy, improving fault diagnosis precision. Experiments on both laboratory bearing faults and industrial check valve faults demonstrate nearly 100% diagnostic accuracy, highlighting the method’s effectiveness and potential.

## 1. Introduction

With the ongoing advancement of industrial technology and the extensive use of mechanical systems, rolling bearings, as essential components, are critical to the performance of industries like the aerospace, high-speed rail, mining, metallurgy, and wind power industries. Over time, various factors can lead to wear and failure of bearings, impacting machinery safety and stability. As a result, effective fault diagnosis methods have become crucial for ensuring equipment reliability and operational efficiency. Among the various approaches, vibration signal analysis has been widely adopted for diagnosing rolling bearing faults.

In rolling bearing fault diagnosis, signal preprocessing and feature extraction are crucial steps. However, traditional time-domain and frequency-domain diagnostic methods face limitations when handling nonlinear and non-stationary signals. To address these challenges, several signal decomposition and feature extraction methods have been developed, improving the accuracy and reliability of bearing fault diagnosis. For instance, Zhu [1] introduced the Improved Singular Value Decomposition Packet (ISVDP) algorithm, which overcomes the limitations in the Hankel matrix structure and enhances feature extraction precision. He [2] proposed the Adaptive Variational Mode Decomposition (AVMD) method, using the Syncretic Impact Index (SII) and the Artificial Bee Colony (ABC) algorithm to optimize the VMD parameters for better transient fault signal extraction. Xiao [3] suggested an intelligent fault diagnosis approach for rotating machinery that integrates Improved VMD (IVMD) with convolutional neural networks (CNNs) to overcome traditional VMD’s reliance on experience-based parameter settings. Lv [4] combined AVMD with optimized Multi-scale Fuzzy Entropy (MFE) to improve the extraction of weak early fault features in complex environments. Xu [5] addressed the issue of weak vibration signals by applying kurtosis and correlation coefficients to filter intrinsic mode functions (IMFs) extracted using Ensemble Empirical Mode Decomposition (EEMD) and reconstructing the signal through the Hilbert transform for fault feature extraction. Ke [6] enhanced Complementary Ensemble Empirical Mode Decomposition (CEEMD) with a genetic algorithm to reduce pattern mixing via adaptive Gaussian white noise adjustment. Zhu [7] proposed a feature fusion approach that combines time-frequency features from the wavelet packet transform with sensitive features extracted via multi-weight singular value decomposition, improving traditional methods’ ability to capture fault features. Mengjiao [8] utilized EEMD to extract multi-domain features from wind turbine bearing signals and used Random Forest (RF) for dimensionality reduction, establishing a fault diagnosis model based on a Deep Belief Network (DBN). Zhang [9] introduced a periodic low-rank dynamic mode decomposition (PLRDMD) method, which improves transient fault feature extraction through periodic window selection and local low-rank approximation, effectively reducing noise interference. Despite these advances, the following challenges remain in dealing with nonlinear and non-stationary rolling bearing signals: (1) mode aliasing between signals leads to the inaccurate selection of IMFs that contain fault information; (2) noise suppression is insufficient, affecting the extraction and analysis of effective signals; and (3) incomplete fault characterization may result in reduced accuracy and understanding of bearing fault conditions.

Feature dimension reduction is essential for handling high-dimensional feature spaces, as it reduces data complexity and enhances the efficiency and performance of classification models. By lowering the number of features, the accuracy and reliability of fault diagnosis can be significantly improved. Traditional dimensionality reduction methods are generally classified into feature selection and feature extraction. Feature selection targets the most relevant features to eliminate redundancy, while feature extraction transforms original data into a lower-dimensional space, preserving key information. Sha [10] introduced the norm-based two-level feature extraction (TFEM) method, which effectively reduces dimensionality by eliminating unimportant features and mitigating the effects of noise and outliers in industrial data. Yang [11] enhanced weak signal feature extraction through improved stochastic resonance, combining amplitude and frequency transform coefficients with a BP neural network and adaptive particle swarm optimization (PSO). Wang [12] employed adaptive frequency shift stochastic resonance and order analysis to convert non-stationary rolling bearing features into steady-state features, thus enhancing the fault feature amplitude and signal-to-noise ratio under variable-speed conditions. Zhong [13] proposed a sparse local Fisher discriminant analysis (SLFDA) model, which retained multiple modes and identified key fault variables, addressing issues in traditional Fisher discriminant analysis. Ying [14] improved the coarse-grained nature of multi-scale permutation entropy (MMPE) through the introduction of composite multivariate multi-scale permutation entropy (CMMPE) and the Laplacian score (LS), which helped avoid information loss. Mochammad [15] combined multi-filter clustering and exhaustive search methods in a multi-filter clustering fusion (MFCF) technique to improve feature selection efficiency and classification accuracy. Zheng [16] extracted complex nonlinear dynamics using clustering feature selection combined with a support vector machine (SVM) optimized by the gravity search algorithm. Ling [17] integrated robust feature selection with K-means clustering into an autoencoder to improve unsupervised feature selection (UFS). Despite these advancements in dimensionality reduction, the following challenges remain: (1) discriminant information is often overlooked during feature selection, reducing effectiveness; (2) noise and outliers in the data still affect algorithm performance; and (3) the robustness and accuracy of feature selection methods require further improvement.

Pattern recognition is crucial in rolling bearing fault diagnosis, as it enables the classification and identification of extracted features, helping engineers detect and address potential equipment failures, thus ensuring safe and reliable machinery operation. Common pattern recognition techniques in fault diagnosis include neural networks, support vector machines (SVMs), and convolutional neural networks (CNNs). Li [18] enhanced the accuracy of balancing machine fault diagnosis by optimizing the Extreme Learning Machine (ELM) with an improved Sparrow Search Algorithm (ISSA), addressing issues of low diagnostic accuracy. Xiao [19] proposed a graph neural network (GNN)-based method for bearing fault detection, which constructed sample similarity maps to improve the identification of low-degree faults that traditional methods fail to capture. In practical engineering applications, equipment faults often involve short-duration events, limited fault samples, and nonlinear distributions, making it difficult for traditional neural networks and deep learning algorithms to handle such complexities, especially when faced with data scarcity. This limitation has led researchers to focus on support vector machines (SVMs) as a more effective tool for diagnosing mechanical failures. However, the application of traditional SVMs in rolling bearing fault diagnosis is hindered by low accuracy and efficiency. Wang [20] developed an SVM kernel function selection mechanism based on sparse representation, applying it to high-speed bearing fault diagnosis to determine the most suitable kernel function type through sparse coding, demonstrating the approach’s superiority. Nevertheless, SVMs face challenges such as high computational complexity and sensitivity to parameter selection, making them less suitable for nonlinear classification problems and large datasets. To improve upon this, Xiong [21] proposed a method combining two-dimensional set local mean decomposition with an optimized dynamic least squares support vector machine (LSSVM), enhancing anti-friction bearing fault diagnosis. Twin support vector machines (TSVMs), which solve two convex quadratic programming (QP) problems and generate non-parallel hyperplanes, offer another alternative. The LSTSVM model, which integrates the advantages of both TSVMs and least squares SVMs, solves linear equations to generate non-parallel hyperplanes, significantly improving computational efficiency. Although these methods have achieved notable results, the following several challenges remain: (1) traditional models perform well with linear data but poorly with nonlinear signals; (2) high computational complexity still limits real-time performance and efficiency; and (3) multi-classification problems remain a challenge that requires further research.

In order to solve the above challenges, this paper proposes a novel fault diagnosis method using FCEEMD-based multi-complexity low-dimensional features and directed acyclic graph LSTSVM, which aims to overcome the existing problems of traditional methods and significantly enhance both the accuracy and efficiency of fault diagnosis. The main contributions of this research are as follows:

(1) The Fast Complementary Ensemble Empirical Mode Decomposition (FCEEMD) method is proposed to decompose the vibration signal into multiple intrinsic mode functions (IMFs). Traditional signal decomposition methods struggle with nonlinear data, often leading to poor signal analysis. By using FCEEMD, the vibration signals are more effectively decomposed, and the reconstructed IMFs are further analyzed using kurtosis and Gini coefficients. This approach significantly improves the accuracy of capturing critical fault information, particularly for nonlinear and non-stationary signals, solving the limitations of previous decomposition techniques.
(2) To tackle the challenges of feature extraction for nonlinear and non-stationary signals, low-dimensional complexity features have become a crucial tool. These features are extracted from high-dimensional signals to effectively represent nonlinear pattern changes while minimizing redundant information, thus better capturing the complexity of signals. To capture multi-level complexities during signal processing, this study introduces multi-complexity features, which provide a comprehensive characterization of signal features across various dimensions and scales, facilitating more precise fault pattern identification. Building on this, this research proposes a novel method for extracting nonlinear complexity features, including sample entropy (SE), permutation entropy (PE), dispersion entropy (DE), Gini coefficient, the squared envelope Gini index (SEGI), and the squared spectral Gini index (SESGI). These complex features are specifically tailored to capture nonlinear characteristics in vibration signals that are often overlooked or inadequately addressed by conventional feature extraction methods. By decomposing signals using the FCEEMD method, we can more efficiently extract these low-dimensional complexity features, reducing noise interference and preserving critical pattern information. Lastly, by integrating these nonlinear features with time-domain and frequency-domain features, a comprehensive high-dimensional feature space matrix is constructed. This integrated approach ensures the preservation of statistical and sensitive nonlinear characteristics, offering a more complete and robust fault feature representation, significantly enhancing the accuracy of fault diagnosis.
(3) The RULSP method is employed to address the issue of high-dimensional data. Traditional feature extraction methods often suffer from feature redundancy and high computational costs, which can hinder diagnostic efficiency. The RULSP method optimizes feature selection by identifying and retaining the most sensitive low-dimensional features while minimizing redundancy. This not only reduces computational complexity but also enhances feature differentiation and sensitivity, ensuring that the selected features are more representative of the fault conditions.
(4) A least squares support vector machine (DAG-LSTSVM) multi-classifier based on a directed acyclic graph is constructed to classify and identify low-dimensional sensitive features. One of the major challenges in fault diagnosis is the multi-classification problem, which is often inadequately handled by traditional classifiers due to high computational requirements and low accuracy in distinguishing between different fault categories. By employing the DAG-LSTSVM model, which combines the strengths of both the least squares SVM and the twin support vector machine (TSVM), the classification process becomes more efficient and accurate. The directed acyclic graph structure further optimizes the classification by efficiently managing multi-class problems, significantly improving fault diagnosis accuracy, even in complex environments.

## 2. Theory and Method

### 2.1. High-Dimensional Feature Extraction Based on FCEEMD

#### 2.1.1. Fast and Complete Ensemble Empirical Mode Decomposition

The Fast and Complete Ensemble Empirical Mode Decomposition (FCEEMD) algorithm is designed to solve the issue of mode aliasing, which is a common problem in traditional empirical mode decomposition (EMD) methods. FCEEMD combines the strengths of both fast EEMD and complete EEMD by improving computational efficiency while reducing mode mixing and reconstruction errors. The key innovation of FCEEMD lies in its ability to systematically suppress mode aliasing through the addition of white noise and iterative decomposition. If a set of vibration signals is $X_{t}=[x_{1},\;x_{2},\ldots ,\;x_{n}]$, the algorithm steps of FCEEMD are as follows:

(1) Initialize the amplitude m and aggregation number I of the added white noise, so that the current aggregation number i=I. This step prepares the algorithm for the iterative addition of white noise that will help to decorrelate the signal components and reduce mode aliasing.
(2) Add i pair of white noise sequences $\pm n_{i}(t),\;i=1,\;2,\ldots ,\;n$ with an equal amplitude and opposite phase (opposite sign) to the original signal x(t), generating two sets of noisy signals $P_{i}(t)$ and $N_{i}(t)$. The noise sequences with opposite phases ensure that the noise introduced in each iteration will cancel each other out when averaged, preserving the true components of the signal.
  (1) $$\left\{\begin{array}{c} P_{i}(t)=x(t)+n_{i}(t)\\ N_{i}(t)=x(t)-n_{i}(t) \end{array}\right.$$
(3) Perform empirical mode decomposition on the signals $P_{i}(t)$ and $N_{i}(t)$ after adding white noise to obtain a series of IMF components.
  (2) $$\left\{\begin{array}{c} P_{i}(t)=\sum_{j=1}^{q}c_{j,i}^{1}(t)\\ N_{i}(t)=\sum_{j=1}^{q}c_{j,i}^{2}(t) \end{array}\right.$$

In the above equations, $c_{j,i}^{1}(t)$ and $c_{j,i}^{2}(t)$ are the *j*-th IMF components obtained by the *i*-th empirical mode decomposition, and q is the number of IMFs.

(4) If the current decomposition degree is less than the maximum decomposition degree (i<I), then increase the decomposition degree by 1 (i=i+1), and then loop through the second and third steps, that is, add white noise sequences $\pm n_{i}(t),\;i=1,\;2,\ldots ,\;n$ with an equal amplitude and opposite phase each time to obtain each IMF and residual R. The new white noise introduced in each new iteration will further decompose the components of the signal while ensuring that modal aliasing is minimized.
(5) Calculate the average of the IMF components obtained through 2I decomposition.
  (3) $$c_{j}(t)=\frac{1}{2I}\sum_{i=1}^{I}\left(c_{j,i}^{1}+c_{j,i}^{2}\right)$$

In the above, $c_{j}(t)$ is the *j*-th IMF obtained by FCEEMD, with an added white noise amplitude of 0.2 and a total of 100 integrations.

The combination of adding white noise, repeated decomposition, and averaging IMFs allows FCEEMD to effectively reduce mode aliasing.

#### 2.1.2. KG Signal Reconstruction for Screening Indicators

(1) Assume that the IMF obtained after FCEEMD decomposition is as follows:

(4) $$FCEEMD(x)=[IMF_{1},IMF_{2},IMF_{3},\ldots ,IMF_{n}]$$

In the above, x is the input vibration signal, and $\left[IMF_{1},\;IMF_{2},\;IMF_{3},\ldots ,\;IMF_{n}\right]$ is the decomposed intrinsic mode function.

(2) Calculate the kurtosis and Gini coefficients of each IMF as follows:

(5) $$\begin{array}{l} Kurtosis(k),Gini(k)\\ k=IMF_{1},\ldots IMF_{n} \end{array}$$

(3) Calculate the KG value of the screening indicator as follows:

(6) KG(k)=Kurtosis(k)∗Gini(k)

(7) $$k=IMF_{1},\ldots IMF_{n}$$

The KG values of all IMFs are sorted, and the important IMFs are selected for subsequent processing.

#### 2.1.3. High-Dimensional Features Based on FCEEMD

(1) Time-domain feature extraction

This paper extracts 16 time-domain features using statistical techniques, with their specific expressions provided in Table 1. Ten features, $TF_{1}\sim TF_{10}$, are known as dimensional statistical parameters, including the mean, standard deviation, square root amplitude, absolute mean, skewness, kurtosis, variance, maximum, minimum, and peak-to-peak values. The remaining six features, $TF_{11}\sim TF_{16}$, consist of the waveform index, peak index, pulse index, edge index, skewness index, and kurtosis index, which are classified as dimensionless statistical parameters. In Table 1, x(n) refers to a discrete time series, and N represents the number of data points in the signal x(n).

(2) Frequency-domain feature extraction

In this paper, for a given time series x(n), 13 frequency-domain features $FF_{2}\sim FF_{6}$ can be extracted by using FFT, and the expressions of these features are described in Table 2. As shown in Table 2, the eigenvalues $FF_{1}$ reflect the vibrational energy in the frequency domain, the eigenvalues $FF_{2}\sim FF_{4}$, $FF_{6}$, and $FF_{10}\sim FF_{13}$ represent the concentration and dispersion of the spectrum, and the eigenvalues $FF_{5}$ and $FF_{7}\sim FF_{9}$ represent the changes in the position of the main frequency bands. Of the thirteen features presented in Table 2, y represents the FFT spectrum for *k* = 1, 2, 3, …, *K*, where *K* denotes the number of spectral lines in the FFT spectrum, and f corresponds to the frequency of the *k*-th spectrum.

(3) Sample entropy feature extraction

Sample entropy is an important nonlinear dynamic feature extraction tool. By assessing the complexity of time series data, it can reveal the health of the bearing. A lower sample entropy indicates that the time series data are more regular, which may reflect the normal operation of the bearings. Conversely, an increase in sample entropy may indicate a failure or increased complexity of the bearing. In bearing fault diagnosis, sample entropy can help identify nonlinear and non-periodic features in the signal, thereby serving as an early warning sign of potential faults. The calculated value of the sample entropy is:

(8) $$S\mathrm{am}pEn(m,r,N)=-\ln(\frac{A_{m}(r)}{B_{m}(r)})$$

*A* represents the total count of forward matches with a length of *m* + 1, while *B* denotes the total count of template matches with a length of m.

(4) Permutation entropy feature extraction

Permutation entropy, as a nonlinear dynamic feature extraction method, identifies the dynamic changes in the system by analyzing the complexity of the time series permutation pattern of the signal. Permutation entropy feature extraction involves arranging adjacent values of a time series to generate a series of permutation patterns and then calculating the frequency distribution of these patterns as signal features. In bearing fault diagnosis, the permutation entropy effectively captures small changes in the condition of the bearing and therefore provides a sensitive indication when the bearing begins to fail. The computational definition of permutation entropy is as follows:

(9) $$PE(T_{1},T_{2})=-\sum_{i=1}^{3!}p_{i}(T_{1}T_{2})\ln p_{i}(T_{1}T_{2})$$

where $p_{i}(T_{1}T_{2})$ represents the relative frequency at which permutations $\pi_{i}$ occur for a given time delay *T_1_* and *T_2_*.

(5) Dispersion entropy feature extraction

Dispersion entropy is an approach used to evaluate the complexity and regularity of vibrational time series data. In contrast to traditional entropy calculation methods, dispersion entropy provides a more precise description of the dynamic behavior and nonlinear traits of a signal by examining its statistical distribution. This method works by segmenting the signal into a series of predefined intervals and determining the frequency of occurrence for each interval along with the degree of dispersion among these frequencies. In the context of bearing fault diagnosis, dispersion entropy is capable of detecting subtle variations in the bearing’s condition, which may be reflected in the frequency distribution, thereby identifying potential fault modes. The process for calculating dispersion entropy is:

(10) $$DE=-\sum_{i=1}^{K}p_{i}\log(p_{i})$$

where $p_{i}$ is the probability of the occurrence of the *i*-th interval data point, and K is the total number of intervals.

(6) Square envelope Gini index feature extraction

The square envelope Gini index is the square envelope applied to time series signals. Square envelope Gini index feature extraction is a method used to analyze the energy distribution uniformity of vibration signals in bearing fault diagnosis. Similar to wavelet energy coefficient feature extraction, the square envelope Gini index reveals the nonlinear characteristics and complexity of a signal by measuring the distribution uniformity of its energy envelope. In bearing fault diagnosis, the square envelope Gini index can identify small changes and early faults in bearings, as faults often lead to significant changes in energy distribution, which are reflected in the Gini index. The square envelope Gini index calculation is defined as follows:

(11) $$SEGI=1-\sum_{k=1}^{N}\left(\frac{2\left|f(k)\right|}{l}\times \frac{N-k+0.5}{N}\right)$$

where N is the length of the signal, f(k) is the *k*-th element of the sorted square envelope spectrum signal $x^{2}$, and l is the sum of the sorted square envelope signals $\left|f\right|$.

(7) Square envelope spectrum Gini index feature extraction

The square envelope spectrum Gini index is applied to the squared envelope spectrum of a time series signal. The role of the square envelope spectrum Gini index in bearing fault diagnosis is to measure the uniformity of the energy distribution of the vibration signal spectrum. By converting the square envelope of a signal into a spectrum and calculating its Gini index, the SESGI provides a method for quantifying the degree of energy concentration in the signal spectrum. In the event of a bearing failure, the spectrum of the signal is often energy-concentrated, with some frequency components being much more energetic than others. The SESGI can effectively capture this energy concentration, making it a useful feature for the early detection and diagnosis of bearing damage status.

(12) $$SESGI=1-2\sum_{j=1}^{L}\left(\frac{\left|x_{j}\right|}{\left\|X\right\|}(\frac{L-j+0.5}{L})\right)$$

where x is the square envelope spectrum of the input signal, $\left|x_{j}\right|$ is the amplitude of the j point in the spectrum, L is the total number of points in the spectrum, and $\left\|x\right\|$ is the sum of the spectral assignments.

(8) Constructing high-dimensional features

The fault features can be extracted from various domains, including the time domain, frequency domain, sample entropy, permutation entropy, dispersion entropy, Gini index, square envelope Gini index, and square envelope spectral Gini index. These features allow for the comprehensive extraction of both state information and the inherent characteristics of the original vibration signal. The following steps outline the high-dimensional feature extraction process:

➀ The sampling frequency is set to be $f_{s}=12000$, the sample length is N=2000, and the samples are divided into $\left[smp_{1},smp_{2},\ldots ,smp_{60}\right]$, a total of 60.

➁ The 16 time-domain features from each $smp_{i}$ sample are extracted, namely:

(13) $$\left[TF_{1},TF_{2},\ldots ,TF_{16}\right]$$

The 13 frequency-domain features are extracted, namely:

(14) $$\left[FF_{1},FF_{2},\ldots ,FF_{13}\right]$$

➂ Each $smp_{i}$ is decomposed using the FCEEMD decomposition method, i.e., each $smp_{i}$ sample is decomposed into $\left[IMF_{1},IMF_{2},\ldots ,IMF_{9}\right]$, the sample entropy feature, permutation entropy feature, dispersion entropy feature, Gini index feature, square envelope Gini index feature, and square envelope spectrum Gini index feature of each $smp_{i}$ is extracted, and they are constructed as time–frequency domain features, that is

(15) $$MDF_{i}=\left[SE_{i},PE_{i},DE_{i},GINI_{i},SEGI_{i},SESGI_{i}\right]$$

➃ The final high-dimensional feature space matrix $GTC_{data}$ is formed by integrating time-domain, frequency-domain, and nonlinear features for each sample. Here, the matrix dimension is 60 × (16 + 13 + 6). The vector $TF_{i\times 16}$ contains 16 time-domain features, and $FF_{i\times 13}$ includes 13 frequency-domain features for the *i*-th sample. Additionally, the sample entropy $SE_{i\times 1}$ permutation entropy $PE_{i\times 1}$, dispersion entropy $DE_{i\times 1}$, Gini coefficient $GINI_{i\times 1}$, square envelope Gini index $SEGI_{i\times 1}$, and square envelope spectral Gini index $SESGI_{i\times 1}$ form the nonlinear feature vector for each sample.

(16) $$GTC_{data}=\left[\begin{array}{c} MDF_{1}\\ MDF_{2}\\ \vdots \\ MDF_{i} \end{array}\right]$$

(17) $$GTC_{data}=\left[\begin{array}{cccccccc} TF_{1\times 16} & FF_{1\times 13} & SE_{1\times 1} & PE_{1\times 1} & DE_{1\times 1} & GINI_{1\times 1} & SEGI_{1\times 1} & SESGI_{1\times 1}\\ TF_{2\times 16} & FF_{2\times 13} & SE_{2\times 1} & PE_{2\times 1} & DE_{2\times 1} & GINI_{2\times 1} & SEGI_{2\times 1} & SESGI_{2\times 1}\\ \vdots & \vdots & \vdots & \vdots & \vdots & \vdots & \vdots & \vdots \\ TF_{60\times 16} & FF_{60\times 13} & SE_{60\times 1} & PE_{60\times 1} & DE_{60\times 1} & GINI_{60\times 1} & SEGI_{60\times 1} & SESGI_{60\times 1} \end{array}\right]$$

### 2.2. Robust Unsupervised Feature Selection with Local Preservation

We introduce a robust unsupervised feature selection method that decomposes the data matrix into two orthogonal components: a parameter matrix and a basis matrix, using matrix factorization techniques. This approach employs 2:1-norm minimization to accurately identify the clustering center, select important features, and preserve the local structure of the original feature space by maintaining local structural terms. The algorithm applies the Alternating-Direction Multiplier Method (ADMM) to optimize the objective function, ensuring robust and efficient feature selection.

Step 1: Define the objective function

The objective function is used to minimize the empirical error by matrix factorization and to select features by sparsity constraints. Specifically, given a data matrix X, which contains n real columns, each instance has a dimension of d, the corresponding dimension of the objective matrix T is m, and the objective function is formalized as:

(18) $$\underset{W,G,F,E}{\min}{\left\|XW-GFT\right\|}_{2,1}+\alpha Tr(W^{T}X^{T}LXW)+k{\left\|W\right\|}_{2,1}$$

In the above, α and K are regularization parameters, L is the Laplacian matrix, and Tr represents the trace of the matrix.

Step 2: Update the W matrix

With the other variables held constant, update the matrix W by solving the following formula:

(19) $$\underset{W}{\min}\alpha Tr(W^{T}X^{T}LXW)+k{\left\|W\right\|}_{2,1}+\frac{\lambda }{2}{\left\|E-XW+GFT-\frac{Y}{\lambda }\right\|}_{F}^{2}$$

Here, λ is the penalty parameter, *E*, *G*, *F*, and *Y* are the other variables, and ${\left\|\cdot \right\|}_{F}^{2}$ is the Frobenius norm.

Step 3: Update the G-matrix

The G-matrix is updated by solving a sub-problem about the *G*-matrix, which is formalized as:

(20) $$\underset{G}{\min}{\left\|GFT-(XW-E+\frac{Y}{\lambda })\right\|}_{F}^{2}\;\;s.t\;G^{T}G=I$$

In Equation (20), I denotes the identity matrix.

Step 4: Update the F-Matrix

The update of the F-matrix is solved by singular value decomposition (SVD). Specifically, given matrices P and Z, the problem to be solved is as follows:

(21) $$\underset{F}{\min}{\left\|P^{T}F-Z\right\|}_{F}^{2}\;\;s.t\;F^{T}F=I$$

Step 5: Update the E-Matrix

The update of E-Matrix is achieved by minimizing the problem about the E-Matrix, which can be formalized as:

(22) $$\underset{E}{\min}{\left\|E\right\|}_{2,1}{\left\|E-(XW-GFT+\frac{Y}{\lambda })\right\|}_{F}^{2}$$

By iterating over the above steps until the convergence conditions are met or the maximum number of iterations is reached, the algorithm ends, and the selected feature set is output. For more details, see ref. [23].

### 2.3. Directed Acyclic Graph Least Squares Twin Support Vector Machine (DAG LSTSVM)

LSTSVM is a variant of the support vector machine (SVM) based on the least squares method for classification tasks. Unlike traditional SVMs, which require solving a convex quadratic programming problem, LSTSVM reformulates the task into solving a system of linear equations by employing equality constraints instead of inequality constraints. This alteration simplifies the solving process. As a result, LSTSVM achieves higher computational efficiency compared to both TSVMs and conventional SVMs while also enhancing classification performance.

(1) Nonlinear least squares twin support vector machine

The objective of LSTSVM is to derive two non-parallel kernel hyperplanes, ensuring that one hyperplane is positioned as close as possible to one class of samples while maintaining distance from the other class. The formulation for the target kernel hyperplane is outlined as follows:

(23) $$K(x^{T},C^{t})w_{1}+b_{1}=0\;\mathrm{and}\;K(x^{T},C^{t})w_{2}+b_{2}=0$$

where $A\in R^{m_{1}\times n}$ is $m_{1}$ positive-class samples, $B\in R^{m_{2}\times n}$ is $m_{2}$ negative-class samples, $K(x^{T},C^{T})$ is the kernel function, C=[A;B] and $w_{i}\in R^{n}$ are hyperplane normal, and $b_{i}$ is biased. The optimization problem of nonlinear LSTSVM is as follows:

(24) $$\begin{array}{l} \underset{w_{1},b_{1},\zeta_{2}}{\min}\frac{1}{2}{\left\|K(A,C^{T})w_{1}+e_{1}b_{1}\right\|}^{2}+\frac{c_{1}}{2}\zeta_{2}^{T}\zeta_{2}\\ s.t.\;-(K(B,C^{T})w_{1}+e_{2}b_{1})+\zeta_{2}=e_{2} \end{array}$$

(25) $$\begin{array}{l} \underset{w_{2},b_{2},\zeta_{1}}{\min}\frac{1}{2}{\left\|K(B,C^{T})w_{2}+e_{2}b_{2}\right\|}^{2}+\frac{c_{2}}{2}\zeta_{1}^{T}\zeta_{1}\\ s.t.\;-(K(A,C^{T})w_{2}+e_{1}b_{2})+\zeta_{1}=e_{1} \end{array}$$

where $c_{1}$ and $c_{2}$ are penalty parameters, $\zeta_{1}$ and $\zeta_{2}$ are relaxation variables, and $e_{1}$ and $e_{2}$ are vectors where each element is 1. By bringing the equality constraints into the objective function, the following unconstrained optimization problems can be obtained:

(26) $$\begin{array}{l} \underset{w_{1},b_{1}}{\min}\frac{1}{2}{\left\|K(A,C^{T})w_{1}+e_{1}b_{1}\right\|}^{2}\\ +\frac{c_{1}}{2}{\left(e_{2}+K(B,C^{T})w_{1}+e_{2}b_{1}\right)}^{T}(e_{2}+K(B,C^{T})w_{1}+e_{2}b_{1}) \end{array}$$

(27) $$\begin{array}{l} \underset{w_{2},b_{2}}{\min}\frac{1}{2}{\left\|K(B,C^{T})w_{2}+e_{2}b_{2}\right\|}^{2}\\ +\frac{c_{2}}{2}{\left(e_{1}-K(A,C^{T})w_{2}-e_{1}b_{2}\right)}^{T}(e_{1}-K(A,C^{T})w_{2}-e_{1}b_{2}) \end{array}$$

If $KerE=[K(A,C^{T})e_{1}]\in R^{m_{1}\times (m+1)}$, $KerF=[K(B,C^{T})e_{2}]\in R^{m_{2}\times (m+1)}$, then the above equations take partial derivatives for $w_{1}$ and $b_{1}$, $w_{2}$ and $b_{2}$, respectively. By constructing a system of linear equations, the solutions to the quadratic programming problem in Equations (24) and (25) can be obtained.

(28) $$v_{+}=-{\left(KerF^{T}\cdot KerF+(1/c_{1})KerE^{T}\cdot KerE\right)}^{-1}\cdot KerF^{T}e_{2}$$

(29) $$v_{-}=-{\left(KerE^{T}\cdot KerE+(1/c_{2})KerF^{T}\cdot KerF\right)}^{-1}\cdot KerE^{T}e_{1}$$

If $KerE=[K(A,C^{T})e_{1}]\in R^{m_{1}\times (m+1)}$, $KerF=[K(B,\;C^{T})e_{2}]\in R^{m_{2}\times (m+1)}$, then in the linear case, the solutions for OPP Equations (26) and (27) can be obtained:

(30) $$(KerE^{T}\cdot KerE+c_{1}KerF^{T}\cdot KerF)\cdot v_{+}=-c_{1}KerF^{T}\cdot e_{2}$$

(31) $$(KerF^{T}\cdot KerF+c_{2}KerE^{T}\cdot KerE)\cdot v_{-}=-c_{2}KerE^{T}\cdot e_{1}$$

where $v_{+}=[w_{1};b_{1}]\in R^{m+1}$, $v_{-}=[w_{2};b_{2}]\in R^{m+1}$; simplifying Equations (30) and (31) leads to:

(32) $$v_{+}=-{\left(KerF^{T}\cdot KerF+(1/c_{1})KerE^{T}\cdot KerE\right)}^{-1}\cdot KerF^{T}e_{2}$$

(33) $$v_{-}=-{\left(KerE^{T}\cdot KerE+(1/c_{2})KerF^{T}\cdot KerF\right)}^{-1}\cdot KerE^{T}e_{1}$$

To solve the nonlinear LSTSVM, it is necessary to compute the inverse of a matrix with dimensions (l+1)(l+1) twice. Subsequently, by applying the Sherman–Morrison–Woodbury (SMW) formula, the solution for the nonlinear LSTSVM can be reduced to calculating the inverse of three matrices, each with dimensions smaller than (l+1)(l+1).

➀ When $m_{1}<m_{2}$, Equations (32) and (33) are rewritten as follows after applying the SMW formula:

(34) $$v_{+}=-(Y-Y\cdot KerE^{T}{\left(c_{1}\cdot I+KerE\cdot Y\cdot KerE^{T}\right)}^{-1}KerE\cdot Y)KerF^{T}e_{2}$$

(35) $$v_{-}=c_{2}(Y-Y\cdot KerE^{T}{\left(I/c_{2}+KerE\cdot Y\cdot KerE^{T}\right)}^{-1}KerE\cdot Y)KerE^{T}e_{1}$$

where $Y={\left(KerF^{T}\cdot KerF\right)}^{-1}$. Drawing on the approach of TSVMs, the regular term εI is introduced into Y, with ε>0, to solve the potential ill-conditioned inverse problem in $KerF^{T}\cdot KerF$.

(36) $$Y=\frac{1}{\varepsilon }(I-KerF^{T}{\left(\varepsilon I+KerF\cdot KerF^{T}\right)}^{-1}KerF)$$

➁ When $m_{2}<m_{1}$, Equations (32) and (33) are rewritten as follows after applying the SMW formula:

(37) $$v_{+}=-c_{1}(Z-Z\cdot KerF^{T}{\left(I/c_{1}+KerF\cdot Z\cdot KerF^{T}\right)}^{-1}KerF\cdot Z)KerF^{T}e_{2}$$

(38) $$v_{-}=(Z-Z\cdot KerF^{T}{\left(c_{2}\cdot I+KerF\cdot Z\cdot KerF^{T}\right)}^{-1}KerF\cdot Z)KerE^{T}e_{1}$$

(39) $$Z=\frac{1}{\varepsilon }(I-KerE^{T}{\left(\varepsilon I+KerE\cdot KerE^{T}\right)}^{-1}KerE)$$

where $Z={\left(KerE^{T}\cdot KerE\right)}^{-1}$; when $m_{1}<m_{2}$, it is necessary to solve the inverse of two $\left(m_{1}\times m_{1}\right)$ matrices and one $\left(m_{2}\times m_{2}\right)$ matrix; when $m_{2}<m_{1}$, it is necessary to solve the inverse of two $\left(m_{2}\times m_{2}\right)$ matrices and one $\left(m_{1}\times m_{1}\right)$ matrix. By solving two systems of linear equations, a pair of solutions for the QPP can be determined. Once $w_{1}$ and $b_{1}$, as well as $w_{2}$ and $b_{2}$, have been obtained, two non-parallel hyperplanes are established. Subsequently, new data points $x\in R^{n}$ are classified into the positive class $W_{1}$ and the negative class $W_{2}$ using the following equation, where $\left|•\right|$ denotes the perpendicular distance from the data point to the kernel hyperplane.

(40) $$x\in W_{k},k=\underset{k=1,2}{\arg\min}\{\left|K(x,C^{T})w_{1}+b_{1}\right|,\left|K(x,C^{T})w_{2}+b_{2}\right|\}$$

(2) The steps of nonlinear LSTSVM are as follows:

Step 1: Enter the positive sample $A\in R^{m_{1}\times n}$ and negative sample $B\in R^{m_{2}\times n}$, and select the appropriate kernel function K.

Step 2: Define $KerE=[K(A,C^{T}),e_{1}]\in R^{m_{1}\times (m+1)}$ and $KerF=[K(B,C^{T}),e_{2}]\in R^{m_{2}\times (m+1)}$.

Step 3: Select the penalty parameters $c_{1}$ and $c_{2}$ through verification.

Step 4: If $m_{1}<m_{2}$, determine the parameters $w_{1}$, $b_{1}$ and $w_{2}$,$b_{2}$ of the two hyperplanes by Equations (34) and (35); otherwise, determine $w_{1}$, $b_{1}$ and $w_{2}$,$b_{2}$ by Equations (37) and (38).

Step 5: Calculate the distance between the new sample $x\in R^{n}$ and the two hyperplanes $\left|K(x,C^{T})w_{1}+b_{1}\right|$ and $\left|K(x,C^{T})w_{2}+b_{2}\right|$, and assign the sample to the positive or negative class by Equation (40). More details are given in [24].

(3) DAG-LSTSVM

The training process of the directed acyclic graph (DAG) requires the generation of K(K−1) non-parallel hyperplanes. The DAG constructs a directed acyclic graph composed of K(K−1)/2 internal nodes and K leaf nodes, and each node is a binary classifier between Class i and Class j. Given the test data, the decision function is evaluated starting from the root node. Depending on the output of the decision function, the test point is shifted to the left or right. The above process is repeated until the test point reaches the leaf node, which can obtain the category of the test point. Finally, based on the minimum distance criterion, the decision functions for Class i and Class j are:

(41) $$d_{ij}=\frac{\left|w_{ij}x+b_{ij}\right|}{\left\|w_{ij}\right\|},d_{ij}=\frac{\left|w_{ji}x+b_{ji}\right|}{\left\|w_{ji}\right\|}$$

If $d_{ij}<d_{ji}$, the distance from the point to Class i is less than the distance to Class j, so it moves in the direction of Class i, that is, j=j−1. The DAG only needs to run (K−1) binary classifiers, and it has the advantages of a short testing time and fewer iterations. At the same time, the DAG solves the problem of the indivisible region of other multi-classification strategies such as OVA, and it has a strong generalization ability and versatility.

## 3. A Novel Fault Diagnosis Method Using FCEEMD-Based Multi-Complexity Low-Dimensional Features and Directed Acyclic Graph LSTSVM

To efficiently and precisely differentiate the types of rolling bearing faults, this study introduces a novel fault diagnosis method using FCEEMD-based multi-complexity low-dimensional features and directed acyclic graph LSTSVM. The procedure is as follows:

(1) First, vibration signals from rolling bearings operating under varying conditions were captured using an accelerometer. The data were sourced from the Case Western Reserve University dataset and an industrial one-way valve dataset. The collected vibration signal was then segmented into 60 non-overlapping segments, each containing 2000 data points.
(2) From each data segment, 16 time-domain features and 13 frequency-domain features were extracted.
(3) The FCEEMD method is proposed to decompose the original vibration signal and generate nine IMFs. It was compared and analyzed with the VMD, EEMD, and CEEMD methods.
(4) Kurtosis (Kurt) and Gini coefficients were calculated for each IMF component to obtain Kurt(k) and Gini(k), where k = 1 to 9. Based on these values, the screening index KG(k) = Kurt(k) × Gini(k) was constructed.
(5) KG(k) was sorted in descending order to obtain the sorted series KG(k)’.
(6) The top four IMF components with the largest values in KG (k)’ were selected for signal reconstruction.
(7) From the reconstructed signal, sample entropy, permutation entropy, and dispersion entropy, along with the Gini coefficient, square envelope Gini coefficient, and square envelope spectral Gini coefficient were extracted to capture nonlinear complexity features.
(8) The extracted time-domain, frequency-domain, and nonlinear complexity features were combined to create a 35-dimensional high-dimensional feature space matrix.
(9) The RULSP method was introduced to identify and select sensitive low-dimensional features from the high-dimensional feature set. Training and test sample sets were generated accordingly. Comparisons were made with other methods, including LS, MCFS, and UDFS.
(10) The DAG-LSTSVM multi-classifier was trained using the training samples, and the resulting model was applied to identify failure modes in the test samples. Comparative analyses were conducted against the SVM, BPNN, ELM, KELM, and DAG-TWSVM methods.

**Figure 1:**
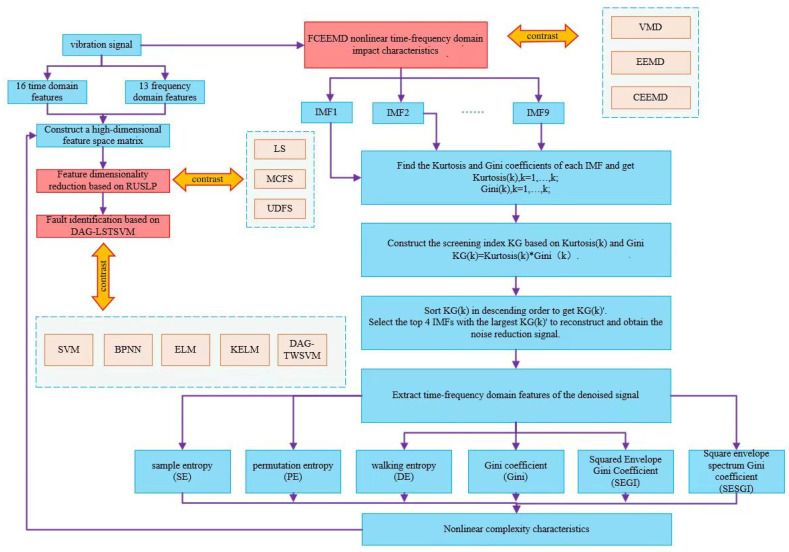
Experimental flowchart.

To demonstrate the effectiveness of the proposed approach, the experimental setup was organized as depicted in the flowchart shown in Figure 1.

## 4. Experimental Verification

### 4.1. Simulation Signal Verification

**Figure 2:**
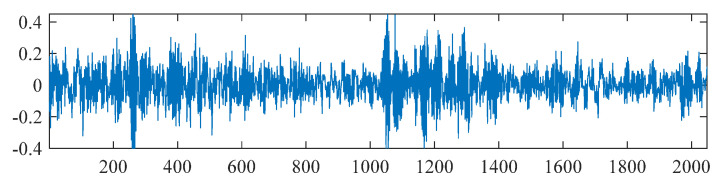
Original signal diagram.

To assess the decomposition performance of the FCEEMD method, we generated an AM-FM signal with a length of 1000, as shown in Equation (42), and introduced white noise at signal-to-noise ratios of 10 dB, 15 dB, 20 dB, and 25 dB. The constructed signal is illustrated in Figure 2.

(42) $$\begin{array}{l} x(t)=[1+0.8\cos(5\pi t)]\cos[150\pi t+1.6\cos(5\pi t)]\\ \;\;+0.5\cos(\pi t)\sin(15\pi t),t\in (0,1) \end{array}$$

**Figure 3:**
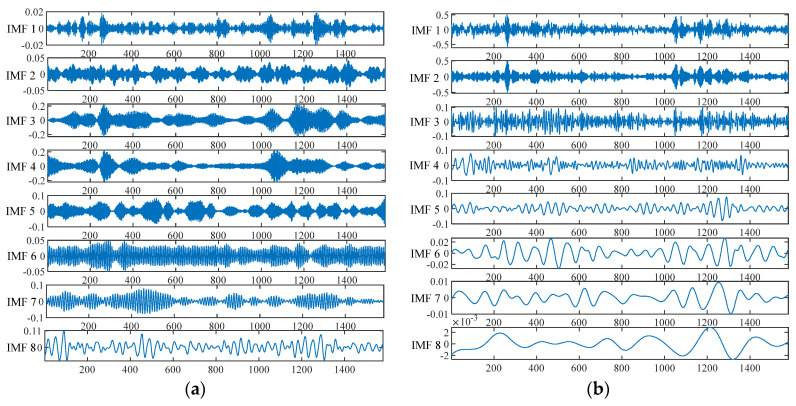

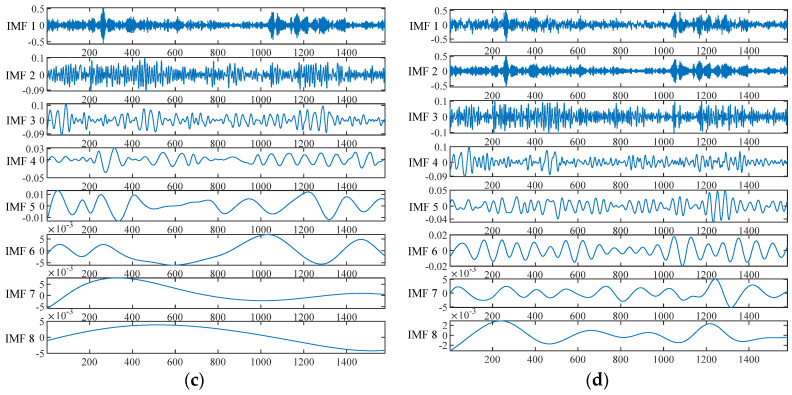
Signal decomposition effects of different methods. (**a**) VMD; (**b**) EEMD; (**c**) CEEMD; (**d**) FCEEMD.

Figure 3 shows a comparison of the effects of the different signal decomposition methods. From the decomposition diagram of VMD shown in Figure 3a, it can be seen that this method could decompose the signal into several relatively smooth modes. However, modal aliasing occurs when processing non-stationary signals, which means that a single mode may contain multiple frequency components, resulting in a decrease in the quality of the decomposition. The EEMD method in Figure 3b aims to reduce modal aliasing by adding white noise to the signal, which helps to capture instantaneous frequency information in the signal. However, additional noise components may be introduced, affecting the decomposition quality. The CEEMD method improves EEMD by adding a series of white noise to the signal to reduce the impact of noise. As shown in Figure 3c, the decomposition diagram shows that CEEMD could generate a more dispersed and clearer IMF, indicating that it has improved compared to EEMD in terms of reducing noise and mode aliasing but with higher computational time complexity. In order to suppress modal aliasing and reduce the reconstruction error, the FCEEMD method adds pairwise white noise with opposite signs to the residual signal of the previous round before each round of signal decomposition. As shown in Figure 3d, the IMF decomposed by FCEEMD was smoother, more discriminative, and more stable in terms of representing local features of the signal, reducing the possibility of noise and mode aliasing. Overall, the FCEEMD method provided a more refined and clear decomposition result, allowing for each component of the signal to be clearly represented.

**Figure 4:**
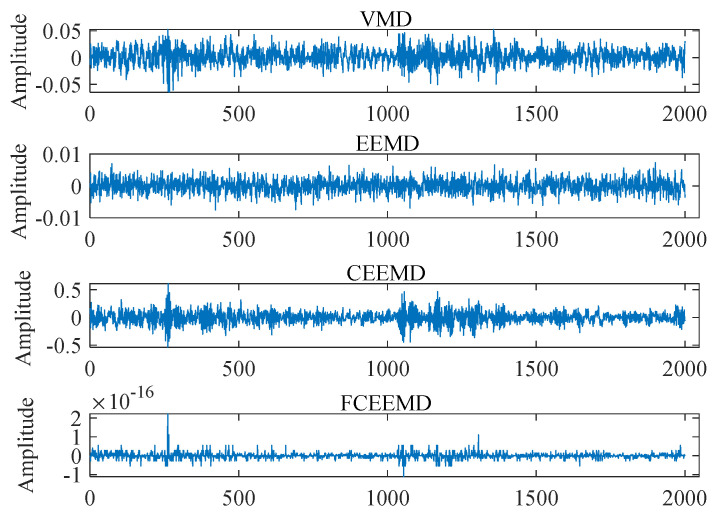
Signal recovery error.

In the field of signal processing, the precise decomposition and reconstruction of signals are of great significance for noise elimination, feature extraction, and further signal analysis. This study delved into four signal decomposition techniques, namely VMD, EEMD, CEEMD, and FCEEMD, and analyzed the recovery errors of each signal decomposition method. The outcome is presented in Figure 4. The recovery error refers to the difference between the original signal and the reconstructed signal of all decomposed IMF components. For signal reconstruction, a smaller error amplitude means that the reconstructed signal is very close to the original signal. In Figure 4, the error amplitude of FCEEMD almost approaches the zero line, indicating that the FCEEMD method hardly lost information from the original signal during the signal reconstruction process. In contrast, the error fluctuations of the other three methods were more pronounced. Especially in some local areas, the fluctuation amplitude was large, indicating that these methods had large errors in the signal reconstruction process in these regions. Due to the complex nonlinearity and non-stationarity of the original signal, the low error of the FCEEMD method suggests that it was better able to handle this complexity.

In order to further quantify the performance of the various signal decomposition methods, indicators such as the reconstruction error, root mean square error (RMSE), decomposition calculation time (Times), and standard deviation (SD) were analyzed. The results are shown in Table 3. The RMSE result of the FCEEMD decomposition method was almost zero (1.3371 × 10^−17^), proving that FCEEMD had high reliability in terms of identifying and separating the essential features of the signals, with almost no signal distortion, and was significantly superior to other comparison methods. The efficiency of the FCEEMD decomposition method was 0.114860 s, which was significantly faster than that of VMD (0.858509 s) and significantly better than that of EEMD (2.508975 s) and CEEMD (4.824789 s). In terms of computing speed, FCEEMD was not only superior to EEMD and CEEMD but also more efficient than VMD, demonstrating its significant advantages in improving computing efficiency. The comparison results of SD further confirm the robustness and consistency of the FCEEMD decomposition method in the signal reconstruction process. Based on the above analysis, the FCEEMD decomposition method demonstrated its superiority in multiple key performance indicators of signal decomposition and reconstruction. It not only had high reconstruction accuracy and fast computational efficiency but also had the smallest standard deviation. These results fully demonstrate the powerful ability of the FCEEMD signal decomposition method, which not only accurately decomposes complex signals but also demonstrated high reliability in reconstructing signals. This ensures that the subsequent extracted features maintain the core information of the signal, which also has a decisive impact on the pattern recognition process.

### 4.2. Case Study on Fault Diagnosis of West Storage Bearings

(1) Experimental platform

**Figure 5:**
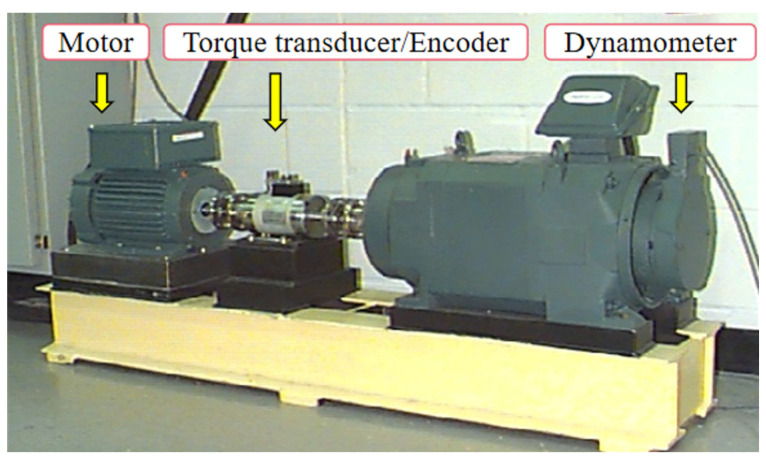
Case Western Reserve University rolling bearing experimental platform [25].

The experimental setup for rolling bearings at Case Western Reserve University is depicted in Figure 5.

The rolling bearing model used was 6205-2RSJEMSKF, with the motor operating at a speed of 1772 rpm. The system’s sampling frequency was set at 12 kHz, and the experimental data length was 2048 points. The details of these parameters are provided in Table 4.

For the experiment, vibration signals were collected from the fan end bearing operating at a motor speed of 1772 r/min. Each group of data consisted of 120,000 sampling points with 2000 points per sample. As a result, each group contained 60 samples, and there were a total of 600 samples across 10 groups of fault data. Among these, samples 1-60 represent normal conditions; samples 61–120, 121–180, and 181–240 correspond to inner ring faults; samples 241–300, 301–360, and 361–420 correspond to outer ring faults; and samples 421–480, 481–540, and 541–600 represent rolling element faults. According to Table 5, the fault diameters for the inner ring, outer ring, and rolling elements were 0.007 inches, 0.014 inches, and 0.021 inches, respectively. The labels for the 10 types of feature vectors were assigned as 1, 2, 3, 4, 5, 6, 7, 8, 9, and 10.

**Figure 6:**
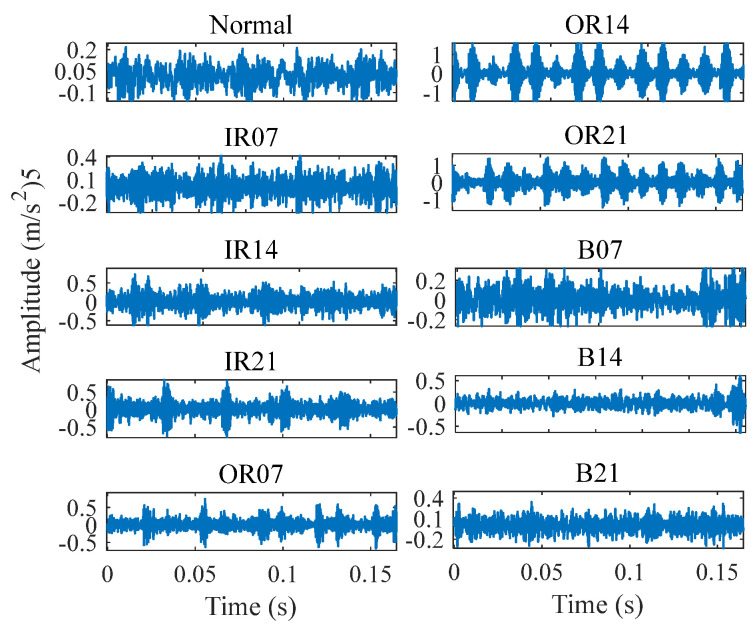
Time-domain waveform of the vibration signal [25].

The time-domain waveform of the vibration signals is illustrated in Figure 6. It is evident that signals IR07, IR21, OR07, OR14, and OR21 exhibited significant periodic impacts, with noticeable differences in amplitude between them. For other signals, the impact characteristics and cycles were less pronounced, indicating the necessity of applying feature extraction techniques and fault diagnosis models to determine the type and severity of bearing faults.

(2) Fault feature extraction

**Figure 7:**
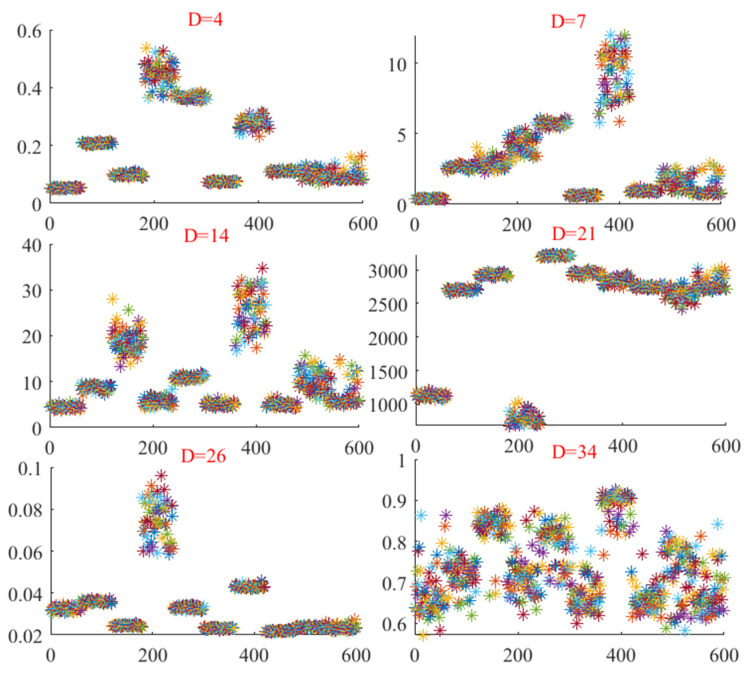
The distribution of feature samples in different dimensions.

The bearing vibration data from the motor at Case Western Reserve University, operating at 1772 r/min, was selected for this experiment. For detailed information about the dataset, please refer to the section on the experimental environment. First, the vibration signals were divided into 60 non-overlapping segments, each with a length of 2000 points. Next, time-domain and frequency-domain features were extracted from each segment of the original vibration signal. To further investigate the time-frequency characteristics of the vibration signals, the IMF components were derived using the FCEEMD method. Subsequently, nonlinear complexity features, including sample entropy, permutation entropy, dispersion entropy, the Gini index, the square envelope Gini index, and the square envelope spectral Gini index, were extracted from the reconstructed signals. The feature distribution of the samples in dimension D is presented in Figure 7.

Figure 7 shows the distribution of ten different state feature samples across six selected dimensions (D = 4, 7, 14, 21, 26, 34). On the D = 4 dimension, the sample points were distributed within a narrow range and overlapped more with each other, indicating that it was very difficult to distinguish the features of the different states in this low dimension. For dimension D = 7, although the distribution range of sample points was expanded, the features of the different states still overlapped, and the boundaries were fuzzy, indicating that even at slightly higher dimensions, the differentiation between features was still insufficient. In the case of D = 14, it can be seen that the sample points began to disperse in a wider space, but the boundaries between the states were still not clear. Concentrated groups appeared at some of the sample points, but the boundaries between groups were not clear. The D = 21 dimension showed a similar pattern, with more scattered sample points, but the boundaries between different states were still blurred. The boundaries between some groups began to appear, but there were still a lot of overlapping areas. In dimension D = 26, although the sample points were more widely distributed in the feature space and the groups formed by each state seemed to be more independent, intersections and overlapping between the groups were still common, which was still not conducive to the clear identification of states. For dimension D = 34, the distribution of the sample points showed higher dispersion, with a slight increase in the space between groups. However, there was still a great deal of overlap, especially in the central region, where the feature groups of multiple states were mixed and difficult to distinguish. On the whole, although the distribution of samples in the feature space gradually dispersed with the increase in dimensions, the feature boundaries of the ten states showed a certain degree of ambiguity in all dimensions. There was no single dimension that clearly separated all the states, indicating a high degree of complexity and similarity between states, which makes feature recognition based on these dimensions more difficult. Therefore, we propose an RUSLP method to reduce the redundancy of features and improve the reliability of fault diagnosis by selecting low-dimensional sensitive features.

(3) Fault feature selection

**Figure 8:**
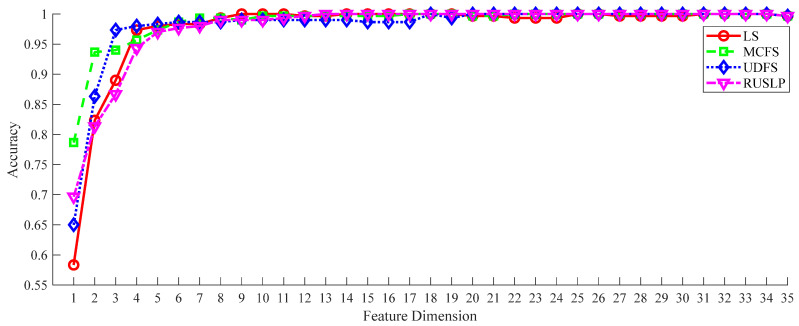
Classification accuracy under different dimensionality reduction methods.

To evaluate the effectiveness of the RUSLP method in detecting bearing faults, a series of experiments were carried out. In these experiments, the FCEEMD decomposition method used in signal processing, as well as the DAG-LSTSVM multi-classifier for classification, remained unchanged. This approach ensured that when RUSLP was compared with the other dimensionality reduction methods, like LS, MCFS, and UDFS, all the methods were applied under identical high-dimensional feature space matrices and parameter configurations. The DAG-LSTSVM multi-classifier parameters were set as follows: OptPara.c1 = c1, OptPara.c2 = c2, OptPara.kerfPara.pars = c3, and OptPara.kerfPara.typ = Kernel Type. The experimental results, illustrating the fault diagnosis accuracy for the different dimensionality reduction methods, are presented in Figure 8.

When the feature dimension was very low (approximately from 1 to 4), all the methods had similar and lower accuracy, indicating that the performance of the classifier was limited in very low dimensions. With the increase in the feature dimension, the accuracy of all methods improved and then stabilized. The RUSLP method exhibited significant performance, achieving high accuracy at lower dimensions and with less fluctuation in accuracy as the dimensions increased. The RUSLP method almost always maintained the highest accuracy throughout the entire dimension range, especially in the range of dimensions from 5 to approximately 30, where the accuracy of the RUSLP method was higher than that of all the other comparison methods. Especially when the number of features was relatively small, the RUSLP method had good performance in terms of bearing fault diagnosis. Although the other dimensionality reduction methods, such as LS, MCFS, UDFS, etc., also exhibited higher accuracy with the increase in the feature dimension, the number of features required to achieve the same level of accuracy exceeded the number of features required for the RUSL method. As shown in Table 6, the RUSLP method could achieve a fault diagnosis accuracy of 99.7% when the feature dimension increased from 1 to 13, and it exceeded the higher dimensions required by the other comparative methods. For example, the LS method needed to achieve 98.7% accuracy in the first 20 dimensions, the MCFS method needed to achieve 96.7% accuracy in the first 15 dimensions, and the UDFS method needed to achieve 99% accuracy in the first 18 dimensions. This indicates that the RUSLP method could not only achieve high accuracy faster but could also achieve this goal in lower dimensions. Overall, the RUSLP method not only performed well in low dimensions but also maintains high accuracy in high dimensions, and it avoided significant performance degradation when the feature dimension was maximized, demonstrating its superior dimensionality reduction ability.

**Figure 9:**
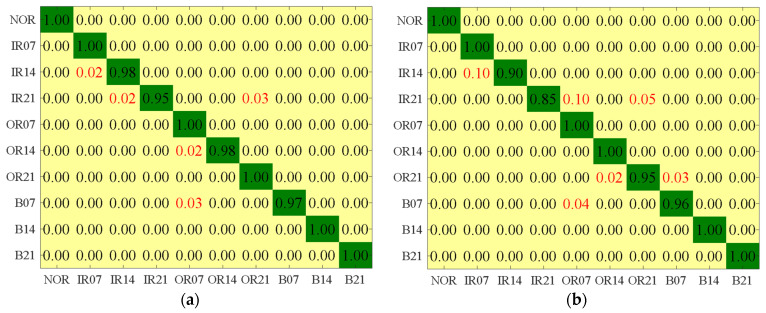

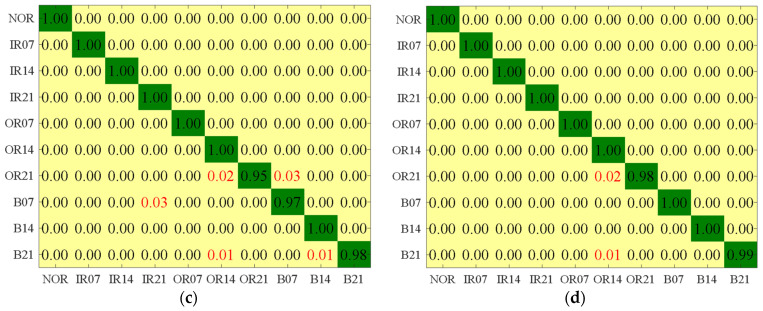
Confusion matrices with the fewest features and highest accuracy obtained by different dimensionality reduction methods. (**a**) LS; (**b**) MCFS; (**c**) UDFS; (**d**) RUSLP.

When the LS, MCFS, UDFS, and RUSLP methods extracted the top 20, top 15, top 18, and top 13 features, respectively, the confusion matrix was obtained and is shown in Figure 9. It can be seen that in terms of feature extraction performance, the experimental results indicate a certain degree of misdiagnosis. Specifically, when using the LS method, 2% of the IR14 states were misclassified as IR07. In addition, the IR21 samples had 2% and 3% false-positive rates diagnosed as IR14 and OR21, while the OR14 and B07 samples had 2% and 3% false-positive rates diagnosed as OR07 and OR14, respectively. The MCFS method had a misdiagnosis rate of up to 10% for diagnosing the IR14 and IR21 samples, and it also showed varying degrees of misjudgment in other categories. Although the UDFS method performed well in most categories, it had a misdiagnosis rate of 2%, 3%, 1%, and 1% in the diagnosis of OR21 to OR14, B07 to IR21, B21 to OR14, and B21 to B14, respectively. In contrast, the RUSLP method showed excellent performance after reducing feature dimensions, with a false-positive rate of only 2% in the diagnosis of OR21 to OR14 and a false-positive rate of 1% in the diagnosis of B21 to OR14. These results highlight the significant advantages of the RUSSLP method in terms of improving the accuracy of bearing fault diagnosis. Compared to other methods, the RUSLP method not only maintained a lower misjudgment rate but also showed higher efficiency in reducing the number of features. Overall, the RUSLP method achieved a high accuracy and low misjudgment rate with fewer feature dimensions, providing an effective solution for mechanical fault diagnosis and demonstrating its significant superiority in practical applications.

(4) Fault identification

**Figure 10:**
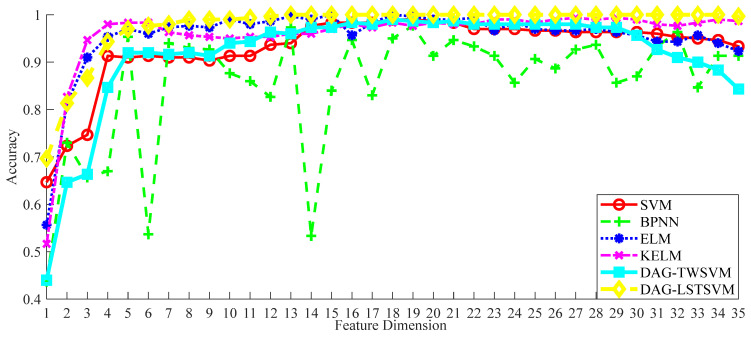
Classification accuracy under different classification methods.

To assess the performance of the DAG-LSTSVM multi-classifier in detecting bearing faults, we conducted a series of experiments. During these tests, the FCEEMD decomposition method for signal processing and the RUSLP algorithm for feature dimensionality reduction remained unchanged. This was carried out to ensure a consistent comparison between the DAG-LSTSVM classifier and the other five classifiers: SVM, BPNN, ELM, KELM, and DAG-TWSVM. All the methods were executed under the same high-dimensional feature space matrix and parameter settings, allowing for a fair evaluation of the effectiveness of DAG-LSTSVM in terms of accurately diagnosing bearing faults. The parameter configurations for the RUSLP method were as follows: X = data matrix, G = encoding matrix, F = orthogonal basis matrix, and W = feature selection matrix. The corresponding comparison results are presented in Figure 10.

The accuracy of all classification methods was indeed low in the 1-to-4-dimensional feature intervals, which may be due to the fact that the features were not sufficient to adequately characterize the fault state within this low-dimensional interval. With the increase in the feature dimension, the accuracy of each classification method was improved and tended to stabilize after a certain point. The accuracy of the DAG-LSTSVM method improved rapidly, and a high classification accuracy can be achieved within a lower-dimensional range, and with the increase in the dimension, its accuracy fluctuated less, showing strong robustness. When looking at the range from 5 to 30 dimensional features, the superiority of the DAG-LSTSVM method is even more obvious. Compared with the other methods, DAG-LSTSVM could achieve high accuracy in lower dimensions. As shown in Table 7, DAG-LSTSVM achieved a classification accuracy of 99.9% on the first 13 dimensional features. In contrast, the other methods required more feature dimensions to achieve a similar level of accuracy. For example, the SVM method achieved a maximum accuracy of about 99% in the first 29 dimensions, while BPNN achieved an accuracy of 97.3% in the first 13 dimensions, the ELM method required an accuracy of 98.7% in the first 15 dimensions, KELM achieved an accuracy of 99.3% in the first 21 dimensions, and DAG-TWSVM achieved an accuracy of 99% in the first 18 dimensions. In general, DAG-LSTSVM showed excellent performance and high accuracy in the application of bearing fault diagnosis, both in low and high dimensions, which fully demonstrates its excellent model generalization ability.

**Figure 11:**
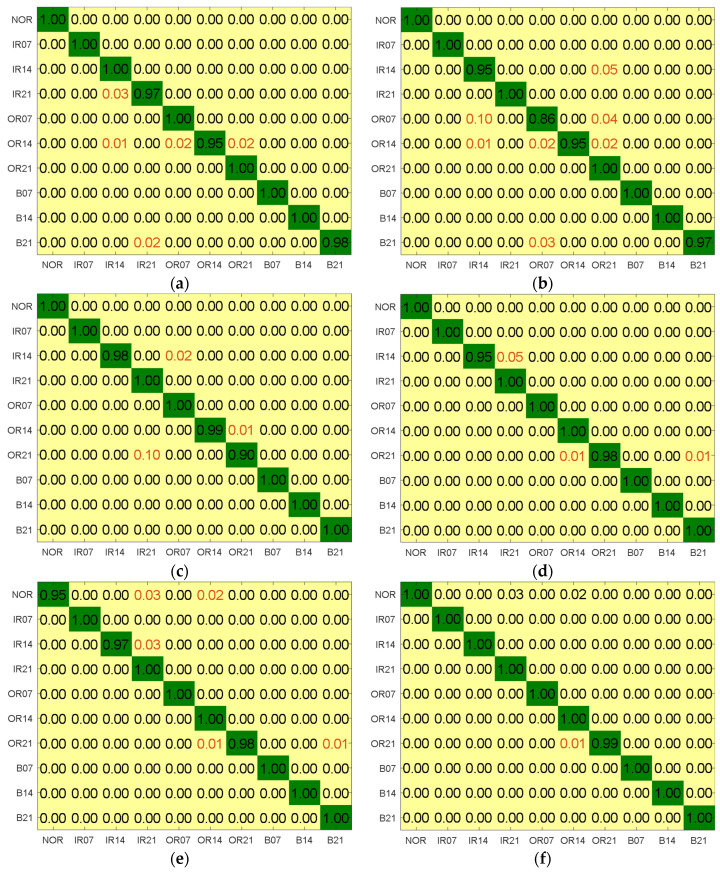
Confusion matrix for different classification methods with the least number of features and the highest accuracy. (**a**) SVM; (**b**) BPNN; (**c**) ELM; (**d**) KELM; (**e**) DAG-TWSVM; (**f**) DAG-LSTSVM.

When the SVM, BPNN, EML, KELM, DAG-TWSVM, and DAG-LSTSVM methods extracted the first 29, 13, 15, 21, 18, and 13 features, respectively, the confusion matrix was obtained and is shown in Figure 11. When using the SVM recognition model, 3% of the IR21 samples were misjudged as the IR14 state, 1% of the OR14 samples were misjudged as the IR14 state, 2% of the OR14 samples were misjudged as the OR07 state, 2% of the OR14 samples were misjudged as the OR21 state, and 2% of the B21 samples were misjudged as the IR21 state. When using the BPNN recognition model, 5% of the IR14 samples were misjudged as the OR21 state, 10% of the OR07 samples were misjudged as the IR14 state, 4% of the OR07 samples were misjudged as the OR21 state, 1% of the OR14 samples were misjudged as the IR14 state, 2% of the OR14 samples were misjudged as the OR07 state, 2% of the OR14 samples were misjudged as the OR21 state, and 3% of the B21 samples were misjudged as the OR07 state. When using the ELM recognition model, 2% of the IR14 samples were misjudged as the OR07 state, 1% of the OR14 samples were misjudged as the OR21 state, and 10% of the OR21 samples were misjudged as the IR21 state. When using the KELM recognition model, 5% of the IR14 samples were misjudged as the IR21 state, 1% of the OR21 samples were misjudged as the OR14 state, and 1% of the OR21 samples were misjudged as the B21 state. When using the DAG-TWSVM recognition model, 3% of the NOR samples were misjudged as the IR21 state, 2% of the NOR samples were misjudged as the OR14 state, 3% of the IR14 samples were misjudged as the IR21 state, and 1% of the OR21 samples were misjudged as the B21 state. It is worth noting that when using the DAG-LSTSVM multi-classifier for recognition, only 1% of the OR21 samples were incorrectly diagnosed as the OR14 state. The comprehensive experimental results show that using the DAG-LSTSVM multi-classifier had a significant effect on improving the accuracy of bearing fault diagnosis. Through a comprehensive comparison of the performance of various models, the results show that the DAG-LSTSVM multi-classifier performed excellently in terms of bearing fault diagnosis accuracy, providing strong support for improving fault diagnosis accuracy.

### 4.3. Diagnosis Experiment for One-Way Valve Faults

(1) Experimental platform

**Figure 12:**
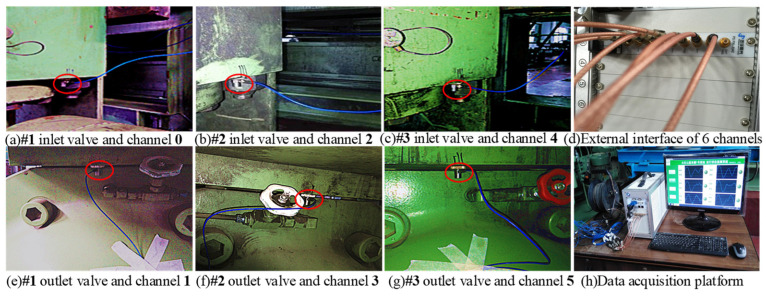
Transmission and sensor measurement point data collection platform.

**Figure 13:**
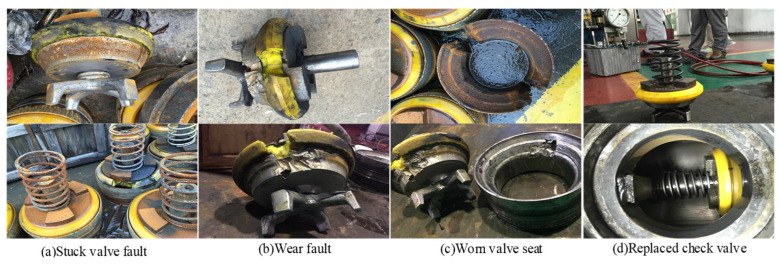
Fault check valve and replaced check valve.

**Figure 14:**
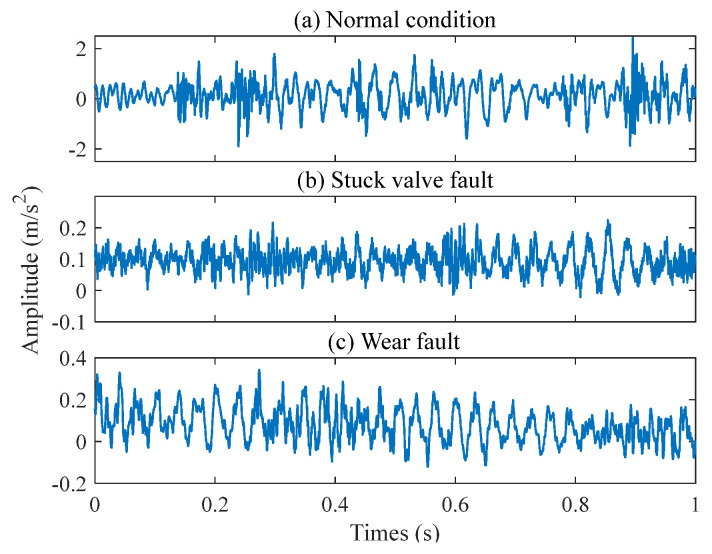
Time-domain waveform of one-way valve vibration signal.

One-way valves function in a challenging slurry transport environment, where the valve core is continually subjected to slurry erosion and the influence of its spring system. As a result, the vibration signal typically consists of fault signals, multi-component vibration coupling signals, and background noise. In this experiment, the vibration signals of these valves were collected during an actual production process, as shown in the sensor setup in Figure 12. Six highly sensitive PCB 352C33 acceleration sensors were installed on the valve housing (marked with a red circle) and connected to a PXI-3342 data acquisition card with eight channels through a high-shielding BNC cable (marked with a blue line). This system captures data at a 24-bit resolution and a top speed of 204.8 KS/s, effectively filtering out noise and converting analog signals to digital. The sampling frequency was set to 2560 Hz, and 76,800 data points were recorded in a single channel, ensuring the quality and detail of the vibration signal. After the signal processing was completed, the data were transferred to a PXI industrial computer and stored under the command of a high-performance PS PXI-3050EXT controller, as shown in Figure 12h. We also captured several common one-way valve faults, including stuck valve faults, wear faults, and wom value seats, as illustrated in Figure 13. Additionally, a scenario of on-site workers replacing valves was demonstrated. The time-domain waveform for a random selection of vibration signals, covering normal operation, stuck valve faults, and wear faults, from the collected data are presented in Figure 14.

(2) Fault feature extraction

**Figure 15:**
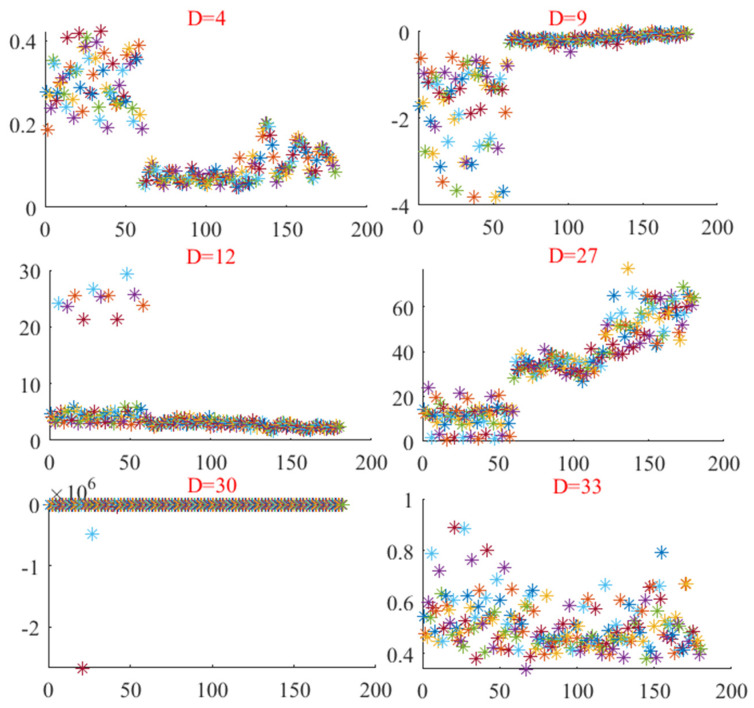
Distribution of feature samples at different dimensions.

The vibration signals from the one-way valve under real operating conditions were utilized as the experimental data, with further data details available in the above section regarding the experimental platform. Initially, the vibration signals under different conditions were divided into 60 distinct, non-overlapping segments, each containing 2000 data points. Next, time-domain and frequency-domain features were extracted from the original bearing vibration signals. To further investigate the time-frequency characteristics of these signals, the IMF components were obtained through the FCEEMD method, and nonlinear complexity features were calculated, including sample entropy, permutation entropy, dispersion entropy, the Gini index, the square envelope Gini index, and the square envelope spectral Gini index from both the analyzed and reconstructed signals. Finally, the time-domain, frequency-domain, and nonlinear complexity features were combined to form a high-dimensional feature space matrix. The distribution of the feature samples across certain dimensions is displayed in Figure 15.

Figure 15 shows the distribution of ten different state feature samples across six selected dimensions (D = 4, 9, 12, 27, 30, 33). On the D = 4 dimension, the feature distribution showed a certain degree of segmentation trend, but there was still significant overlapping between groups. This indicates that at low dimensions, although some states began to show signs of separation, their recognizability was not high. In the distribution of D = 9 dimensions, the sample points began to form a linear trend, but the boundaries between states remained blurred, especially in the horizontal distribution where there was almost no clear separation. In dimension D = 12, the distribution of the sample points was relatively flat, with most sample points occurring near the horizontal axis and a few points being distributed above, indicating that most states were difficult to distinguish in this dimension. A relatively scattered distribution was displayed in the D = 27 dimension, and the sample points formed a clear trend line, indicating that some states started to separate in this dimension, but there was still a problem of crossing and overlap among the features. In the D = 30 dimension, the sample points almost completely overlapped and compressed on a straight line, with almost no identification information between states. The D = 33 dimension presents a more complex situation, where although the distribution between states was more dispersed, there was still a lot of overlap, making state recognition challenging. In summary, although the different dimensions exhibited different distribution characteristics, there was a common problem of blurred boundaries between states and difficulty in identifying them clearly. Although some dimensions exhibited certain trends or distribution patterns, no single dimension could provide clear distinctions for all states. Therefore, we propose the RUSLP method, which sorts and selects mixed features to remove features with low correlation, thereby reducing feature redundancy and improving the reliability of fault diagnosis.

(3) Fault feature selection

**Figure 16:**
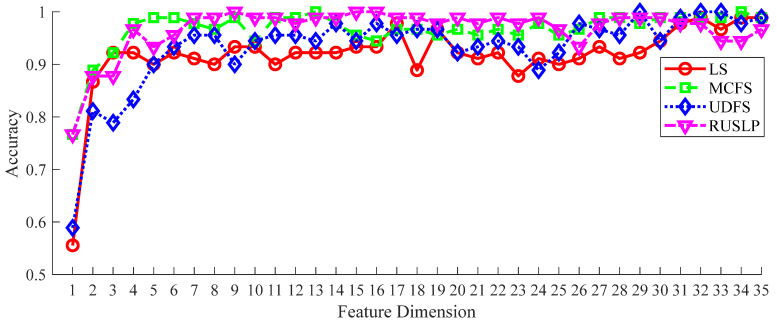
Classification accuracy under different dimensionality reduction methods.

In order to verify the effectiveness of the RUSLP method in terms of bearing fault detection, a series of experiments were conducted. In the experiment, we kept the FCEEMD decomposition method in the signal processing step unchanged and the DAG-LSTSVM multi-classifier in the classification recognition step unchanged. This was carried out to ensure that when comparing RUSLP with the other dimensionality reduction methods, i.e., LS, MCFS, and UDFS, all the methods were carried out under the same high-dimensional feature space matrix and parameter settings. As a result, we could more fairly evaluate the effectiveness of RUSLP in terms of accurately diagnosing bearing faults. The parameter configuration of the DAG-LSTSVM multi-classifier was consistent with the above. Figure 16 shows the fault diagnosis accuracy results of each decomposition method.

In the first to the fourth dimensional feature intervals, the classification accuracy of all the dimensionality reduction methods was low, indicating that the performance of the classification model was limited in this extremely low dimensional interval, and the characteristics were not sufficient to characterize the fault state. However, with the increase in the feature dimension, the classification accuracy of each method improved and gradually stabilized. In this process, the performance of the RUSLP method was particularly outstanding, which shows that it could achieve a high classification accuracy in the lower dimension range, and the accuracy fluctuation was small in the process of increasing the dimension. Compared with the LS, MCFS, UDFS, and other methods, the RUSLP method needed fewer feature dimensions to achieve similar or even better classification results when the features were in the range of 5 to 30 dimensions. As shown in Table 8, the RUSLP method achieved 100% classification accuracy in the first nine dimensions, while other methods required more feature dimensions to achieve a similar level of accuracy. For example, the LS method achieved 97.8% accuracy in the first 17 dimensions, the MCFS method achieved 100% accuracy in the first 12 dimensions, and the UDFS method required the first 29 dimensions to achieve 98% accuracy. Based on the above analysis, the RUSLP method not only had a significant effect in the low dimensions but also maintained a high accuracy in the higher dimensions. These characteristics highlight the dimensionality reduction ability of the RUSLP method and its potential to improve diagnostic accuracy in terms of bearing fault diagnosis, especially in application scenarios with limited feature dimensions, where its advantages are particularly evident.

**Figure 17:**
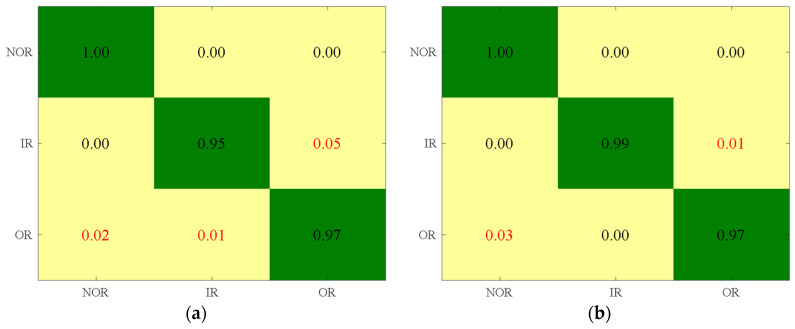

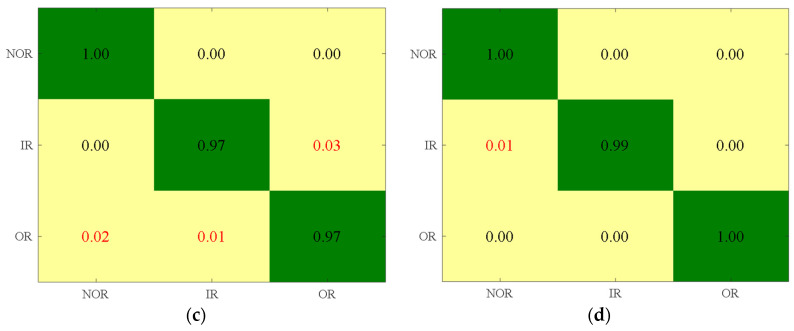
Confusion matrices with the least number of features and the highest accuracy obtained by different dimensionality reduction methods. (**a**) LS; (**b**) MCFS; (**c**) UDFS; (**d**) RUSLP.

When applying the LS, MCFS, UDFS, and RUSLP methods, they extracted the first 17, 12, 29, and 9 dimensions, respectively. The corresponding confusion matrix is shown in Figure 17. When the LS dimensionality reduction method was applied, 5% of the IR samples were misclassified as OR, 2% of the OR samples were misclassified as NOR, and 1% of the OR samples were misclassified as IR. When using the MCFS method, 1% of the IR samples were misclassified as OR, and 3% of the OR samples were misclassified as NOR. For the UDFS method, 3% of the IR samples were misclassified as OR, 2% of the OR samples were misclassified as NOR, and 1% of the OR samples were misclassified as IR. With the RUSLP method, 1% of the IR samples were misclassified as NOR. Based on the comparison of the performance across the different dimensionality reduction methods, the findings highlight the superior accuracy of the RUSLP method in bearing fault diagnosis and underscore its significant contribution to improving diagnostic accuracy.

(4) Fault identification

**Figure 18:**
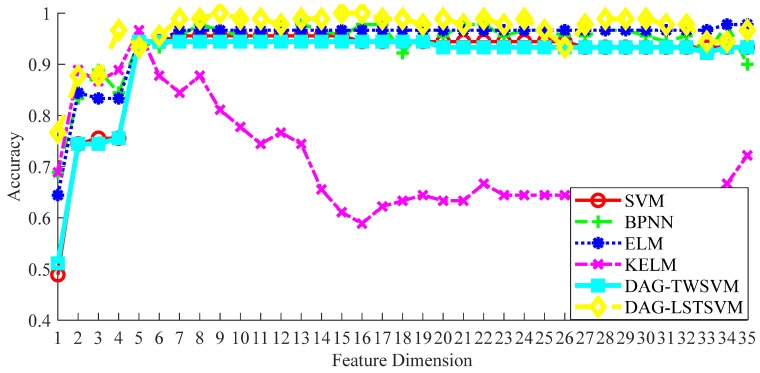
Classification accuracy under different classification methods.

In order to evaluate the performance of the DAG-LSTSVM multi-classifier for bearing fault detection, a series of experiments were conducted. In these experiments, we kept the FCEEMD decomposition method unchanged in the signal processing step and the RUSLP algorithm unchanged in the feature dimensionality reduction step. This was carried out to ensure that when comparing the DAG-LSTSVM classifier with the other five classifiers, i.e., SVM, BPNN, ELM, KELM, and DAG-TWSVM, all the methods were carried out under the same high-dimensional feature space matrix and parameter settings. As a result, we could fairly evaluate the effectiveness of the DAG-LSTSVM method in terms of accurately diagnosing bearing faults. Figure 18 shows the accuracy of the fault diagnosis of each method.

The accuracy of all the classification methods was generally low in the first to fourth dimensional feature intervals, which indicates that the features in the very-low-dimensional interval did not adequately represent the fault state. However, with the increase in the feature dimension, the classification accuracy improved and gradually stabilized. Among all the methods, DAG-LSTSVM showed its superiority. As shown in Figure 18, the performance of DAG-LSTSVM showed a high classification accuracy in the lower dimension range, and the accuracy fluctuation was small in the process of increasing the feature dimension, indicating that the method had strong robustness. When observing the range from 5 to 30 dimensional features, DAG-LSTSVM required fewer feature dimensions to achieve better classification results than SVM, BPNN, ELM, KELM, and DAG-TWSVM. As shown in Table 9, DAG-LSTSVM achieved 100% classification accuracy in the first nine dimensions, while the other methods required more feature dimensions to achieve a similar level of accuracy. For example, the SVM method achieved a maximum accuracy of 95.6% in the first 7 dimensions, BPNN achieved an accuracy of 97.8% in the first 13 dimensions, ELM achieved an accuracy of 96.7% in the first 34 dimensions, KELM achieved an accuracy of 96.7% in the first 5 dimensions, and DAG-TWSVM achieved an accuracy of 94.5% in the first 5 dimensions. In summary, DAG-LSTSVM not only performed well in the low dimensions in terms of bearing fault diagnosis, but it also maintained a high accuracy in the high dimensions, indicating that DAG-LSTSVM has a good model generalization ability.

**Figure 19:**
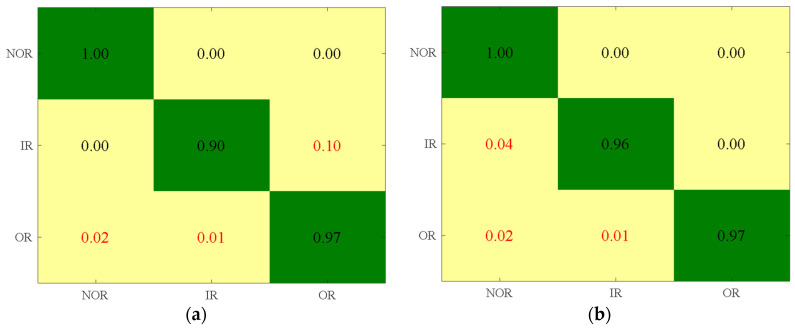

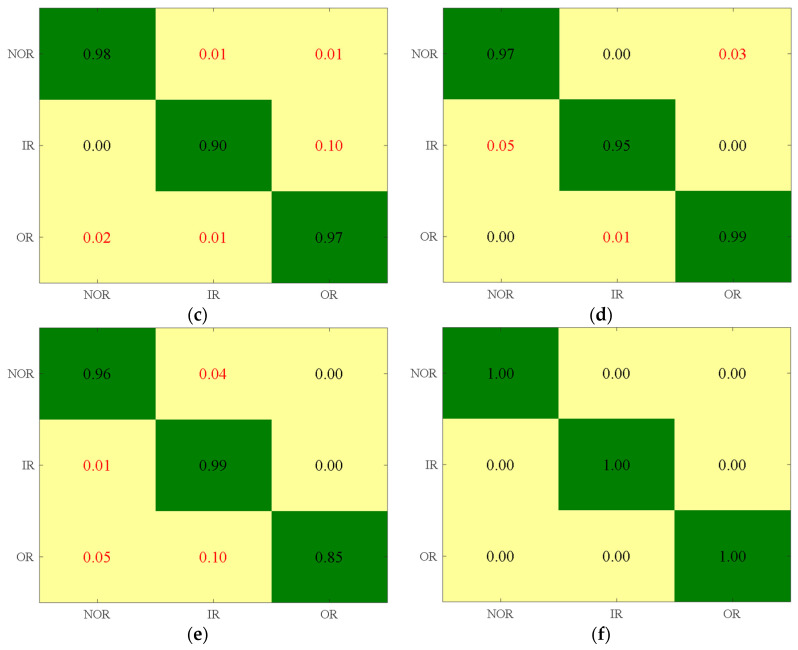
Confusion matrix for different classification methods with the least number of features and the highest accuracy. (**a**) SVM; (**b**) BPNN; (**c**) ELM; (**d**) KELM; (**e**) DAG-TWSVM; (**f**) DAG-LSTSVM.

When applying the SVM, BPNN, EML, KELM, DAG-TWSVM, and DAG-LSTSVM methods, they extracted the features from the first 7, 13, 34, 5, and 9 dimensions, respectively. The corresponding confusion matrix is illustrated in Figure 19. When the SVM model was used, 10% of the IR samples were misclassified as OR, 2% of the OR samples were misclassified as NOR, and 1% of the OR samples were misclassified as IR. When the BPNN model was employed, 4% of the IR samples were misclassified as NOR, 2% of the OR samples were misclassified as NOR, and 1% of the OR samples were misclassified as IR. For the ELM model, 1% of the NOR samples were misclassified as IR, 1% were misclassified as OR, and 5% of the IR samples were misclassified as NOR. Using the KELM model resulted in 3% of the NOR samples being misclassified as OR, 5% of the IR samples were misclassified as NOR, and 1% of the OR samples were misclassified as IR. With the DAG-TWSVM model, 4% of the NOR samples were misclassified as IR, 1% of the IR samples were misclassified as NOR, 5% of the OR samples were misclassified as NOR, and 10% of the OR samples were misclassified as IR. In contrast, the DAG-LSTSVM multi-classifier accurately identified all three states. The experimental findings demonstrate that the DAG-LSTSVM multi-classifier significantly enhances bearing fault diagnosis accuracy.

## 5. Conclusions

Due to the presence of different types of frequency interference in bearing vibration signals, traditional methods often fail to deliver satisfactory results. To address the limitations of previous fault diagnosis techniques, this paper introduces a new fault diagnosis method based on FCEEMD multi-complexity low-dimensional features and directed acyclic graph LSTSVM. Bearing failure data from the Case Western Reserve University laboratory, along with check valve failure data from industrial environments, were analyzed in terms of feature extraction, feature selection, and classification identification. These analyses demonstrated the effectiveness and superiority of the proposed method. The key conclusions are as follows:

(1) Compared with the VMD, EEMD and CEEMD methods, the proposed FCEEMD method can more accurately decompose the IMFs from the vibration signal and construct the screening coefficient by the kurtosis and Gini coefficients to effectively analyze and reconstruct these IMFs. This process not only significantly improves the noise reduction ability but can also suppress the interference noise in the signal without losing key fault information, and it also improves the efficiency of nonlinear data processing and significantly enhances the fault feature identification ability.
(2) Compared with the traditional feature extraction methods in the time domain and frequency domain, we propose a new multi-dimensional feature extraction method that involves extracting sample entropy (SE), permutation entropy (PE), dispersion entropy (DE), the Gini coefficient, the square envelope Gini coefficient (SEGI), and the square envelope spectral Gini coefficient (SESGI) in the reconstructed signal to construct nonlinear complexity features and combining them with the features in the time domain and frequency domain to obtain a comprehensive and high-dimensional feature space matrix. In the high-dimensional feature space matrix, sample entropy is very effective for revealing sudden changes in and impact characteristics of fault signals. Permutation entropy and dispersion entropy can extract the complexity of and dynamic changes in the signal, which is helpful in terms of identifying the periodic characteristics of the signal. The Gini coefficient and square envelope Gini system can accurately reflect the irregularity and fault impact characteristics of signals. The Gini coefficient of the square envelope spectrum further provides a measure of the inhomogeneity of the signal energy distribution in the frequency domain and helps to identify the specific frequency components caused by faults. This method not only solves the shortcomings of traditional methods in nonlinear time-frequency feature extraction but also provides a possibility for comprehensive fault mode identification through the construction of a high-dimensional feature space matrix, ensures the effective acquisition of various fault features, and shows advantages in dealing with impacts and periodic features.
(3) Compared with the traditional LS, MCFS and UDFS feature selection methods, the RUSLP method obtains low-dimensional sensitive features through a fine feature selection process, significantly improving the distinguishing ability and sensitivity of features, effectively reducing the number of required features and ensuring the quality of these features. This not only solves the common problem of feature redundancy in traditional methods but also greatly reduces the computational complexity and improves the efficiency and accuracy of fault diagnosis.
(4) Compared with the SVM, BPNN, EML, KEML and DAG-TWSVM classification methods, the proposed DAG-LSTSVM multi-classifier not only successfully overcomes the challenge of multi-classification problems but also improves the accuracy and accuracy of diagnosis when fault diagnosis is performed on a feature set after dimensionality reduction. The experimental results show that in both the Case Western Reserve University laboratory dataset and the industrial environment check valve dataset, the proposed method achieved a nearly 100% fault diagnosis accuracy.
(5) In summary, the method proposed in this study shows significant advantages and potential in the field of bearing fault diagnosis and demonstrates promising application prospects. However, the experiments were conducted on specific datasets from laboratory and industrial environments, which may not fully capture the diversity of real-world conditions. Therefore, the generalization of the method across more complex and diverse industrial environments requires further validation. Future research will focus on enhancing the generalization ability of the method and optimizing its computational efficiency to better adapt to variable industrial environments and handle more complex failure modes.

## Figures and Tables

**Table 1:** Expression of time-domain characteristic parameters [22].

Feature Expression		
$TF_{1}=\frac{1}{N}\sum_{n=1}^{N}x(n)$	$TF_{2}=\sqrt{\frac{1}{N-1}\sum_{n=1}^{N}{\left[x(n)-TF_{1}\right]}^{2}}$	$TF_{3}={\left(\frac{1}{N}\sum_{n=1}^{N}\sqrt{\left|x(n)\right|}\right)}^{2}$
$TF_{4}=\frac{1}{N}\sum_{n=1}^{N}\left|x(n)\right|$	$TF_{5}=\frac{1}{N}\sum_{n=1}^{N}{\left(x(n)\right)}^{3}$	$TF_{6}=\frac{1}{N}\sum_{n=1}^{N}{\left(x(n)\right)}^{4}$
$TF_{7}=\frac{1}{N}\sum_{n=1}^{N}{\left(x(n)\right)}^{2}$	$TF_{8}=\max\left|x(n)\right|$	$TF_{9}=\min\left|x(n)\right|$
$TF_{10}=TF_{8}-TF_{9}$	$TF_{11}=\frac{TF_{2}}{TF_{4}}$	$TF_{12}=\frac{TF_{8}}{TF_{2}}$
$TF_{13}=\frac{TF_{8}}{TF_{4}}$	$TF_{14}=\frac{TF_{8}}{TF_{3}}$	$TF_{15}=\frac{TF_{5}}{{\left(\sqrt{TF_{7}}\right)}^{3}}$
$TF_{16}=\frac{TF_{6}}{{\left(TF_{7}\right)}^{2}}$		

**Table 2:** Expression of frequency-domain characteristic parameters [22].

Feature Expression		
$FF_{1}=\frac{\sum_{k=1}^{K}y(k)}{K}$	$FF_{6}=\sqrt{\frac{\sum_{k=1}^{K}\left[{\left(f_{k}-FF_{5}\right)}^{2}y(k)\right]}{K}}$	$FF_{11}=\frac{\sum_{k=1}^{K}\left[{\left(f_{k}-FF_{5}\right)}^{3}y(k)\right]}{K{\left(FF_{6}\right)}^{3}}$
$FF_{2}=\frac{{\sum_{k=1}^{K}\left[y(k)-FF_{1}\right]}^{2}}{K-1}$	$FF_{7}=\sqrt{\frac{\sum_{k=1}^{K}\left(f_{k=1}^{K}y(k)\right)}{\sum_{k=1}^{K}y(k)}}$	$FF_{12}=\frac{\sum_{k=1}^{K}\left[{\left(f_{k}-FF_{5}\right)}^{4}y(k)\right]}{K{\left(FF_{6}\right)}^{4}}$
$FF_{3}=\frac{\sum_{k=1}^{K}{\left[y(k)-FF_{1}\right]}^{3}}{K{\left(\sqrt{FF^{2}}\right)}^{3}}$	$FF_{8}=\sqrt{\frac{\sum_{k=1}^{K}\left(f_{k}^{4}y(k)\right)}{\sum_{k=1}^{K}\left(f_{k}^{2}y(k)\right)}}$	$FF_{13}=\frac{\sum_{k=1}^{K}\left[\sqrt{\left|f_{k}-FF_{5}\right|}y(k)\right]}{K\sqrt{FF_{6}}}$
$FF_{4}=\frac{\sum_{k=1}^{K}{\left[y(k)-FF_{1}\right]}^{4}}{K{\left(FF_{2}\right)}^{2}}$	$FF_{9}=\frac{\sum_{k=1}^{K}\left(f_{k}^{2}y(k)\right)}{\sqrt{\left[\sum_{k=1}^{K}\left(f_{k}^{4}y(k)\right)][\sum_{k=1}^{K}y(k)\right]}}$	—
$FF_{5}=\frac{\sum_{k=1}^{K}\left(f_{k}y(k)\right)}{\sum_{k=1}^{K}y(k)}$	$FF_{10}=\frac{FF_{6}}{FF_{5}}$	—

**Table 3:** Comparison of decomposition indicators.

Decomposition Method	RMSE	Time	SD
VMD	0.0209	0.85851	0.1011
EEMD	0.0023	2.50898	0.1108
CEEMD	0.1109	4.82479	0.2218
FCEEMD	1.3371 × 10^−17^	0.11486	0.1009

**Table 4:** Structural parameters of SKF 6205 bearings [25].

Bearing Type	Rolling Element Diameter	Number of Rolling Elements	Bearing Pitch Diameter	Contact Angle
SKF 6205	7.938 mm	9	39 mm	0

**Table 5:** Time-domain waveform of the vibration signal [25].

Category	Size	Abbreviation	Label
Normal	0.000 inches	Normal	1
Failure of inner ring	0.007 inches	IR07	2
	0.014 inches	IR14	3
	0.021 inches	IR21	4
Failure of outer ring	0.007 inches	OR07	5
	0.014 inches	OR14	6
	0.021 inches	OR21	7
Failure of roller	0.007 inches	B07	8
	0.014 inches	B14	9
	0.021 inches	B21	10

**Table 6:** The highest accuracy and corresponding feature dimension x, obtained by different dimensionality reduction methods.

Dimensionality Reduction Model	LS	MCFS	UDFS	RUSLP
Front X-dimensional features	20	15	18	13
Highest precision	98.7%	96.7%	99%	99.7%

**Table 7:** The highest accuracy obtained by different classification methods and the corresponding feature dimension x.

Classification Model	SVM	BPNN	ELM	KELM	DAG-TWSVM	DAG-LSTSVM
Front X dimensional features	29	13	15	21	18	13
Maximum accuracy	99%	97.3%	98.7%	99.3%	99%	99.9%

**Table 8:** The highest accuracy and corresponding feature dimension x, obtained by different dimensionality reduction methods.

Dimensionality Reduction Model	LS	MCFS	UDFS	RUSLP
Front X-dimensional features	17	12	29	9
Maximum accuracy	97.8%	98.9%	98%	99.3%

**Table 9:** The highest accuracy and corresponding feature dimension x, obtained by different classification methods.

Classification Model	SVM	BPNN	ELM	KELM	DAG-TWSVM	DAG-LSTSVM
Front X-dimensional features	7	13	34	5	5	9
Maximum accuracy	95.6%	97.8%	96.7%	96.7%	94.5%	100%

## Data Availability

This experiment is still under study and the data will not be disclosed for the time being.
